# Supplementary material for: Orthogonal intercellular signaling for programmed spatial behavior
Source: Mol Syst Biol. 2016 Jan 26;12(1):849. doi: 10.15252/msb.20156590 (PMC4731010; doi:10.15252/msb.20156590)
Supplement: Supplementary file 1 — Appendix [file MSB-12-849-s001.pdf]

Appendix to
Orthogonal intercellular signaling for programmed spatial behaviors

Paul K. Grant[1,2,*], Neil Dalchau[2,*], James Brown[1,2], Fernan Federici[1], Tim Rudge[1], Boyan Yordanov[2], Om Patange[1], Andrew Phillips[2], and Jim Haseloff[1]

[1]Department of Plant Sciences, University of Cambridge, Cambridge, CB2 3EA, UK
[2]Computational Science Laboratory, Microsoft Research, Cambridge, CB1 2FB, UK
[*]These authors contributed equally

Contents

A Supplementary experimental details **2**
- A.1 Plasmid details . . . 2
- A.2 Comparison of *standard* promoters . . . 3
- A.3 Summary of experimental data . . . 3

B Full model **4**
- B.1 Derivation from chemical reactions . . . 4
- B.2 Non-dimensionalisation . . . 6
- B.3 Empirical alteration of the "Hill coefficient" . . . 7
- B.4 Inducible expression of LuxR/LasR . . . 7
- B.5 Parameter inference . . . 8
- B.6 Model-data comparison . . . 11

C A simplified model that has a closed-form solution **13**
- C.1 Derivation of the transfer function . . . 13
- C.2 The functional response to combinations of C_6 and C_{12} . . . 14
- C.3 Parameter inference . . . 15

D Characterization using GEC **18**

E Reducing crosstalk **21**
- E.1 Model-based determination of optimal LuxR and LasR levels . . . 21
- E.2 Characterization of double receivers . . . 21

F Modelling spatio-temporal dynamics resulting from HSL diffusion **24**
- F.1 Characterization of HSL diffusion and sender cells . . . 24
- F.2 Re-characterizing the pLas81 response . . . 26
- F.3 Characterization of relay sender devices . . . 27
- F.4 Simulation of relay cells in a checkerboard arrangement . . . 30

A Supplementary experimental details

A.1 Plasmid details

Table S1: Ratiometric reporter constructs. All plasmids built on backbone pSB2K3 with an added reference promoter (all pR except pR0011LL123* which is Bba_J23101) driving eCFP [1, 2]. All RBS sequences are Bba_B0034.

Plasmid name	Promoter driving receiver protein(s)	Receiver proteins expressed	Promoter driving eYFP
pR0011LL123	pLlacO-1 (Bba_R0011)	LuxR, LasR	pLux (Bba_R0062)
pR0011LL123*	pLlacO-1 (Bba_R0011)	LuxR, LasR	pLux (Bba_R0062)
pCatLL123	pCat (Bba_I14033)	LuxR, LasR	pLux (Bba_R0062)
pCatR123	pCat (Bba_I14033)	LuxR	pLux (Bba_R0062)
pCatS123	pCat (Bba_I14033)	LasR	pLux (Bba_R0062)
pCatLL76	pCat (Bba_I14033)	LuxR, LasR	pLux76
pCatR76	pCat (Bba_I14033)	LuxR	pLux76
pCatS76	pCat (Bba_I14033)	LasR	pLux76
pCatLL81	pCat (Bba_I14033)	LuxR, LasR	pLas81
pCatR81	pCat (Bba_I14033)	LuxR	pLas81
pCatS81	pCat (Bba_I14033)	LasR	pLas81
pBADLuxR	pBAD (BBa_I0500)	LuxR	N/A
pBADLasR	pBAD (BBa_I0500)	LasR	N/A

Table S2: Double receiver constructs. All plasmids built on backbone pSB2K3 with pLas81 driving eYFP and pLux76 driving eCFP both with RBS Bba_B0034. The asterisk symbol indicates synthetic RBS designed using the RBS calculator [3].

Plasmid name	LuxR promoter	LuxR RBS	LasR promoter	LasR RBS
pCatR34S34	pCat	Bba_B0034	pCat	Bba_B0034
pR33S34	Bba_R0040	Bba_B0033	Bba_R0011	Bba_B0034
pR33S175	Bba_R0040	Bba_B0033	Bba_R0011	S175*
pR33S32	Bba_R0040	Bba_B0033	Bba_R0011	Bba_B0032
pR100S34	Bba_R0040	S100*	Bba_R0011	Bba_B0034
pR100S32	Bba_R0040	S100*	Bba_R0011	Bba_B0032

Table S3: Sender constructs. The asterisk symbol indicates synthetic RBS designed using the RBS calculator [3].

Plasmid name	Promoter	RBS	CDS	Origin/antibiotic
pR0011LuxI	Bba_R0011	Bba_B0034	LuxI	P15a/Kan
pBADLasI	PBAD	Bba_B0034	LasI	P15a/Kan
pLux76LasI	PLux76	S900*	LasI	pMB1/Amp
pLas81LuxI	PLas81	Bba_B0032	LuxI	pMB1/Amp

A.2 Comparison of *standard* promoters

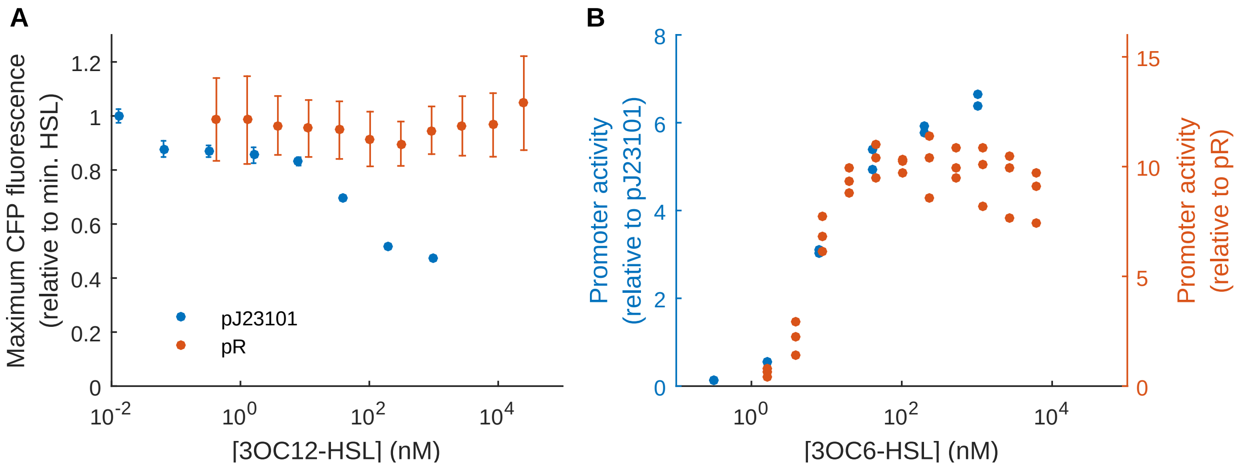

Figure S1: pR, unlike Bba_J23101, is not inhibited by C12-LasR but serves as an equivalent reference promoter. (**A**) Normalized activity (deCFP/dOD normalized to activity at [C12]=0) of reference promoter pR (orange) or Bba_J23101 (blue) in the presence of high expression of receiver proteins (pR0011LL123, pR0011LL123*), as a function of 3OC12-HSL concentration. (**B**) Ratiometric activity (deYFP/deCFP) of pLux (Bba_R0062) with respect to reference promoter pR (orange, pR0011LL123) or Bba_J23101 (blue, pR0011LL123*), as a function of 3OC6-HSL concentration.

A.3 Summary of experimental data

Here, we summarize the experimental measurements that were used for inferring parameters of the models. Five different strategies were used to probe the response of pLux promoters to HSL signal concentration, each providing a different control over the intracellular level of LuxR and LasR protein. These are detailed in Table S4.

Table S4: Datasets used for characterizing pLux promoters. For descriptions of each plasmid, see Table S1.

**Plasmids**	**Number of datasets**		
	$k = 123$	$k = 76$	$k = 81$
pCatLL*k*	3	5	3
pCatR*k*	5	3	4
pCatS*k*	4	3	2
pBADLuxR + pCatS*k*	3	3	3
pBADLasR + pCatR*k*	3	3	3

B Full model

B.1 Derivation from chemical reactions

As introduced in the main text, the model is based on the following chemical reactions:

$$
\begin{array}{rcl|rcl}
\varnothing & \xrightarrow{n_R} & R & \varnothing & \xrightarrow{n_S} & S \\
R + C_k & \underset{u_{Rk}}{\overset{b_{Rk}}{\rightleftharpoons}} & R_k & S + C_k & \underset{u_{Sk}}{\overset{b_{Sk}}{\rightleftharpoons}} & S_k \\
R_k + R_k & \underset{u_{Dk}}{\overset{b_{Dk}}{\rightleftharpoons}} & D_k & S_k + S_k & \underset{u_{Ek}}{\overset{b_{Ek}}{\rightleftharpoons}} & E_k \\
G + D_k & \underset{u_{GDk}}{\overset{b_{GDk}}{\rightleftharpoons}} & G.D_k & G + E_k & \underset{u_{GEk}}{\overset{b_{GEk}}{\rightleftharpoons}} & G.E_k \\
G.D_k & \xrightarrow{a_{Rk}} & G.D_k + \text{mRNA} & G.E_k & \xrightarrow{a_{Sk}} & G.E_k + \text{mRNA} \\
G & \xrightarrow{a_0} & G + \text{mRNA} & \varnothing & \xrightarrow{n_G} & G \\
\multicolumn{3}{l}{R, S, R_k, S_k, D_k, E_k, G, G.D_k, G.E_k \xrightarrow{\gamma} \varnothing} & & &
\end{array}
\tag{1}
$$

where R represents LuxR, S represents LasR, C_k represents HSL of type k (either 6 or 12). By specifying the rate constants as distinct in this way, we cover all possibilities of the various interactions being signal-specific, LuxR/LasR-specific, etc. For example, it may or may not be important to model promoter-binding of D_k or E_k as being dependent on whether the regulators incorporate AHL_6 or AHL_{12} signals. If not, we would simply enforce $b_{GD6} = b_{GD12}$ and $b_{GE6} = b_{GE12}$. We assume a zero-order production rate for gene G, which models the replacement of plasmids during cell division. This is motivated by wanting to balance plasmid replication with dilution in equilibrium. Finally, we note that transcription and translation of LuxR/LasR are lumped into a single generation reaction. This is for simplicity, as we will seek an equilibrium eventually, and the LuxR/LasR factors in the model will become subsumed into a single parameter.

Translating the reaction system (1) to a system of differential equations, assuming mass action kinetics, we obtain

$$\frac{d[R]}{dt} = n_R + u_{Rk}[R_k] - [R](\gamma + b_{Rk}[C_k]) \tag{2a}$$

$$\frac{d[R_k]}{dt} = b_{Rk}[R][C_k] + 2u_{Dk}[D_k] - [R_k](\gamma + u_{Rk} + 2b_{Dk}[R_k]) \tag{2b}$$

$$\frac{d[D_k]}{dt} = b_{Dk}[R_k]^2 + u_{GDk}[G.D_k] - [D_k](\gamma + u_{Dk} + b_{GDk}[G]) \tag{2c}$$

$$\frac{d[G]}{dt} = n_G + u_{GDk}[G.D_k] - (\gamma + b_{GDk}[D_k])[G] \tag{2d}$$

$$\frac{d[G.D_k]}{dt} = b_{GDk}[G][D_k] - (\gamma + u_{GDk})[G.D_k] \tag{2e}$$

There will also be an equivalent set of equations for LasR, which can be appended to describe cells expressing both receiver proteins.

Ideally, we would seek a closed-form expression for the steady-state transcription from gene G in cells expressing only LuxR (we return to the multiple receiver proteins case later), by equating the equations (2) to zero. By considering conservation of mass for LuxR and G, we note that

$$0 = \frac{d[R]}{dt} + \frac{d[R_k]}{dt} + 2\left(\frac{d[D_k]}{dt} + \frac{d[G.D_k]}{dt}\right) = n_R - \gamma\left([R] + [R_k] + 2.[D_k] + 2.[G.D_k]\right) \tag{3a}$$

$$0 = \frac{d[G]}{dt} + \frac{d[G.D_k]}{dt} = n_G - \gamma\left([G] + [G.D_k]\right) \tag{3b}$$

By defining a series of equilibrium constants

$$[R_k] = K_{Rk}[R][C_k], \quad [D_k] = K_{Dk}[R_k]^2, \quad [G.D_k] = K_{GRk}[G][D_k], \tag{4}$$

then substituting into (3), we obtain

$$\frac{n_R}{\gamma} = \left([R] + K_{Rk}[R][C_k] + 2(1 + K_{GRk}[G])K_{Dk}K_{Rk}^2[R]^2[C_k]^2\right) \tag{5a}$$

$$\frac{n_G}{\gamma} = [G]\left(1 + K_{GRk}K_{Dk}K_{Rk}^2[R]^2[C_k]^2\right) \tag{5b}$$

Eqn. (5b) can be rearranged to give an expression

$$[G] = \frac{n_G}{\gamma\left(1 + K_{GRk}K_{Dk}K_{Rk}^2[R]^2[C_k]^2\right)}, \tag{6}$$

which can be substituted into (5a) to give

$$\frac{n_R}{\gamma} = \left([R] + K_{Rk}[R][C_k] + 2\left(1 + \frac{n_G K_{GRk}}{\gamma\left(1 + K_{GRk}K_{Dk}K_{Rk}^2[R]^2[C_k]^2\right)}\right)K_{Dk}K_{Rk}^2[R]^2[C_k]^2\right) \tag{7}$$

Therefore, to obtain a complete solution, it remains to solve (7) numerically for $[R]$, and then substitute into (4) and (6). Note that this equation is quartic in $[R]$, and so an algebraic solution may be formulated, but it is too complex to extract any useful interpretation.

The rate of mRNA synthesis is given by $f_{Ck} = a_0[G] + a_1[G.D_k]$, which after substituting the above expressions can be rearranged to give the model output as

$$f_{Ck} = \left(a_0 + a_{Rk}K_{GRk}K_{Dk}K_{Rk}^2[R]^2[C_k]^n\right)[G] \tag{8}$$

where $[G]$ and $[R]$ are as determined above.

Multiple receiver proteins. When LasR is present and expressed at rate n_S, (6) becomes

$$[G] = \frac{n_G}{\gamma\left(1 + K_{GRk}K_{Dk}K_{Rk}^2[R]^2[C_k]^2 + K_{GSk}K_{Ek}K_{Sk}^2[S]^2[C_k]^2\right)} \tag{9}$$

The solution for $[R]$ and $[S]$ are then obtained via numerical solutions in the same way as before. The transcription rate f_{Ck} is determined analogous to the above.

Multiple promoters in competition. Finally, to extend to the case of n competing promoters each produced at rate n_G^j and with LuxR/LasR affinities K_{GRk}^j and K_{GSk}^j for $j \in \{1, \ldots, n\}$, we obtain the solution for each unbound promoter as

$$[G_j] = \frac{n_G^j}{\gamma\left(1 + K_{GRk}^j K_{Dk}K_{Rk}^2[R]^2[C_k]^2 + K_{GSk}^j K_{Ek}K_{Sk}^2[S]^2[C_k]^2\right)} =: g_j([R]) \tag{10}$$

Accordingly, the concentrations $[R]$ and $[S]$ are obtained by numerically solving

$$\frac{n_R}{\gamma} = [R](1 + K_{Rk}[C_k]) + 2\left(1 + \sum_j K_{GRk}^j \cdot g_j([R])\right)K_{Dk}K_{Rk}^2[R]^2[C_k]^2 \tag{11a}$$

$$\frac{n_S}{\gamma} = [S](1 + K_{Sk}[C_k]) + 2\left(1 + \sum_j K_{GSk}^j \cdot g_j([R])\right)K_{Ek}K_{Sk}^2[S]^2[C_k]^2 \tag{11b}$$

B.2 Non-dimensionalisation

Since we are interested in the transcription rate from gene G, which is given by $a_0[G] + a_1[G.D_k]$, we can consider the rescaling of $[G] \to \alpha G$. Equivalently, we can rescale the level of LuxR in the system, i.e. $[R] \to \beta R$. Using similar scales on the complexes, $[R_k] \to \beta R_k$, $[D_k] \to \beta D_k$ and $[G.D_k] \to \beta.GD_k$.

With these rescalings, the conservation equations (3) become

$$0 = n_R - \gamma\left(\beta R + \beta R_k + 2\beta.D_k + 2\beta.GD_k\right) \tag{12}$$

$$0 = n_G - \gamma\left(\alpha.G + \beta.GD_k\right) \tag{13}$$

By setting $\alpha = \dfrac{n_G}{\gamma}$ and $\beta = \dfrac{n_R}{\gamma}$, then defining K_{Rk}, K_{Dk} and K_{GRk} as before, we obtain

$$G = \frac{1}{1 + \rho.K_{GRk}.K_{Dk}K_{Rk}^2 R^2 C_k^2} \tag{14}$$

where $\rho = \frac{\beta}{\alpha}$. Therefore, R is the solution to

$$1 = \left(1 + K_{Rk}C_k\right).R + 2\left(1 + \frac{K_{GRk}}{1 + \rho.K_{GRk}.K_{Dk}K_{Rk}^2 R^2 C_k^2}\right) K_{Dk}K_{Rk}^2 C_k^2.R^2 \tag{15}$$

The non-dimensionalized transcription rate is as in (8).

Multiple receiver proteins. When LasR is present the non-dimensionalized equivalent of (9) becomes

$$G = \frac{1}{1 + \rho_R.K_{GRk}K_{Dk}K_{Rk}^2 R^2 C_k^2 + \rho_S.K_{GSk}K_{Ek}K_{Sk}^2 S^2 C_k^2} \tag{16}$$

where $\rho_R = \frac{n_R}{n_G}$ and $\rho_S = \frac{n_S}{n_G}$. The solution for R and S are then obtained numerically as before.

Multiple promoters in competition. The non-dimensionalized equivalent of (10) is

$$G_j = \frac{1}{1 + \rho_R^j K_{GRk}^j K_{Dk}K_{Rk}^2 R^2 C_k^2 + \rho_S^j K_{GSk}^j K_{Ek}K_{Sk}^2 S^2 C_k^2} =: \hat{g}_j(R) \tag{17}$$

Accordingly, the non-dimensionalized concentrations R and S are obtained by numerically solving

$$1 = R\left(1 + K_{Rk}C_k\right) + 2\left(1 + \sum_j \hat{g}_j(R)\right) K_{Dk}K_{Rk}^2 R^2 C_k^2 \tag{18a}$$

$$1 = S\left(1 + K_{Sk}C_k\right) + 2\left(1 + \sum_j \hat{g}_j(R)\right) K_{Ek}K_{Sk}^2 S^2 C_k^2 \tag{18b}$$

Note, we drop the superscript j on the ρ_R and ρ_S parameters as the same plasmid is used for expressing LuxR and LasR in our experiments.

In this form, the parameterisation is extremely general. There are different rates of transcription depending on whether LuxR or LasR-based tetramers are bound, and also whether a C_6 or C_{12} molecule is bound. To ensure that the parameters are sufficiently constrained by the data, it is often necessary to make simplifying assumptions, that may well be representative of the underlying biochemistry. For instance, we expect that whether the tetramer contains C_6 or C_{12} molecules will not affect recruitment of RNA polymerase (and therefore $a_{R6} = a_{R12} = a_R$, $a_{S6} = a_{S12} = a_S$). The same may also be true of the binding of tetramer to the promoter. Whereas the binding of C_6 to LuxR will be different to the binding of C_{12} to LuxR, and similarly for LasR, though the homo-binding of LuxR-C_6 dimers (for example) may or may not be signal-specific. The question is how much signal and receiver specificity is required in the reaction parameters in order to reproduce multiple experimental measurements.

B.3 Empirical alteration of the "Hill coefficient"

The more common approach to modeling HSL-based signaling is to use a Hill function representation $f(c)$ for the transcriptional dependency on HSL concentration c []. For example,

$$f(c) = \frac{a_0 + a_1 K^n c^n}{1 + K^n c^n} \tag{19}$$

where a_0 is the basal transcription rate, a_1 is the transcription rate of the bound promoter, K is the *sensitivity* (reciprocal of the half-maximal saturating concentration) and n is the *Hill coefficient*. One would expect n to take on values near 2, in line with there being 2 HSL molecules in the regulator of the promoter. However, empirically, this value is often inferred to be a lower value.

In our models with more complex transfer functions, we find that the exponent 2 that arises in the underlying equations leads to responses that are too steep with respect to the HSL concentration. Therefore, we applied a correction to the model that allowed a free parameter n that is analogous to the Hill coefficient. As such, the full model becomes

$$1 = R\left(1 + K_{Rk} C_k^{\frac{n}{2}}\right) + 2\left(1 + \sum_j \frac{1}{1 + \rho_R K_{GRk}^j K_{Dk} K_{Rk}^2 R^2 C_k^n + \rho_S K_{GSk}^j K_{Ek} K_{Sk}^2 S^2 C_k^n}\right) K_{Dk} K_{Rk}^2 R^2 C_k^n \tag{20a}$$

$$1 = S\left(1 + K_{Sk} C_k^{\frac{n}{2}}\right) + 2\left(1 + \sum_j \frac{1}{1 + \rho_R K_{GRk}^j K_{Dk} K_{Rk}^2 R^2 C_k^n + \rho_S K_{GSk}^j K_{Ek} K_{Sk}^2 S^2 C_k^n}\right) K_{Ek} K_{Sk}^2 S^2 C_k^n \tag{20b}$$

B.4 Inducible expression of LuxR/LasR

Experiments were performed in which cells expressing one receiver protein under the control of the constitutive expresser pcat promoter and the other receiver protein under the control of the arabinose-inducible pbad promoter. For convenience, we assume that pcat expression of LuxR mRNA results in LuxR protein at concentration 1. i.e. we work in *pcat units* r representing the relative concentration of LuxR. We describe the control of r via arabinose-pbad relative to this value.

To model the quantity of LuxR/LasR synthesized via the pbad promoter, we used a Hill function, as has been done previously [4]. Note, the Hill function can also be obtained from rearranging more detailed representations, such as in [5, 6]. We used the parameterization

$$f_A([\text{Ara}]) = \frac{a_1^A.[\text{Ara}] + a_0^A.K_A}{[\text{Ara}] + K_A} \tag{21}$$

Additionally, we allowed the eventual LasR versus LuxR levels at arabinose concentration A to differ by a constant scale factor. As such, we assign $s = s_{\text{bad}}.f_A([\text{Ara}])$.

We also alternatively tried inferring a separate parameter for each different concentration of arabinose. However, the inferred values nicely coincided with the Hill function representation, so we opted to show results corresponding to the simpler Hill functional scheme.

B.5 Parameter inference

The parameters of the full model were inferred using Filzbach. Eight independent MCMC chains were run with 125,000 burn-in iterations and 250,000 samples. The majority of the marginal posterior distributions were convincingly Gaussian-shaped (Fig. S2), though some parameters were poorly identifiable, such as ρ_R, ρ_S and K_{GR}. Evidence of poor identifiability of the same parameters could also be seen in the correlation structure of the joint posterior (Fig. S3). The value of n was convincingly less than 2, which helps to explain the poorer performance we observed when n was fixed equal to 2 (not shown).

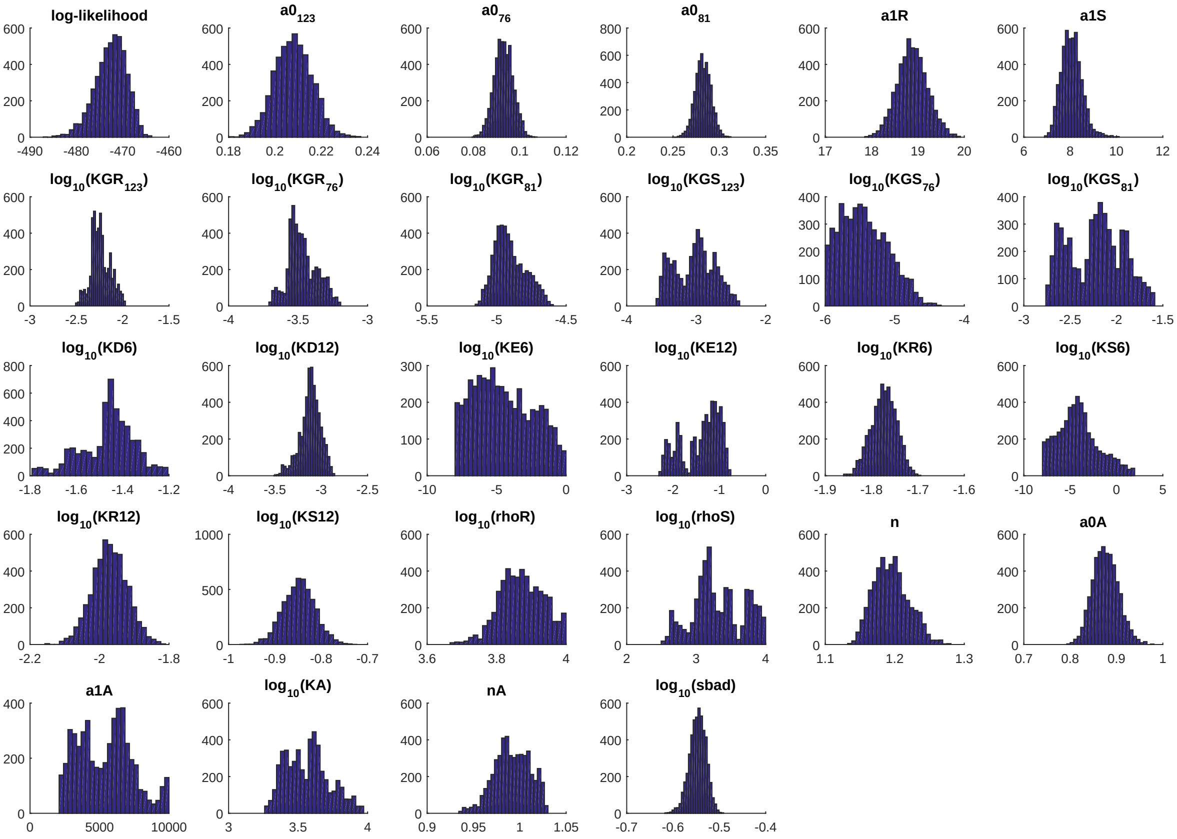

Figure S2: Marginal posterior parameter distributions for the full model. The marginal parameter posterior distributions are shown for each inferred parameter in the model. Parameters for which the base-10 logarithm is shown are those parameters which used a logarithmic proposal distribution, while other parameters had real-valued proposals. The histograms shown represent every 50th sample out of a total of 250,000 samples in a single MCMC chain. The maximum log-likelihood score attained was -472.6.

Table S5: Parameters Summary. Each parameter of the full model is defined, along with its maximum likelihood estimate (MLE). For parameter inference, all prior distributions were uniform, with the ranges specified below. The proposal distributions for some parameters were set to a logarithmic scale, as indicated below.

Parameter	Description	MLE	Prior	Scale
a_0^{123}	Basal transcription rate of P_{Lux}	0.208	$[0, 10]$	Real
a_0^{76}	Basal transcription rate of P_{OLux}	0.093	$[0, 10]$	Real
a_0^{81}	Basal transcription rate of P_{OLas}	0.282	$[0, 10]$	Real
a_1^R	Transcription rate with LuxR regulators bound	18.881	$[0, 100]$	Real
a_1^S	Transcription rate with LasR regulators bound	8.067	$[0, 100]$	Real
K_{GR}^{123}	Affinity of P_{Lux} for LuxR regulators	5.80×10^{-3}	$[10^{-6}, 10^2]$	Log
K_{GR}^{76}	Affinity of P_{OLux} for LuxR regulators	3.40×10^{-4}	$[10^{-6}, 10^2]$	Log
K_{GR}^{81}	Affinity of P_{OLas} for LuxR regulators	1.25×10^{-5}	$[10^{-6}, 10^2]$	Log
K_{GS}^{123}	Affinity of P_{Lux} for LasR regulators	9.49×10^{-4}	$[10^{-6}, 10^2]$	Log
K_{GS}^{76}	Affinity of P_{OLux} for LasR regulators	3.66×10^{-6}	$[10^{-6}, 10^2]$	Log
K_{GS}^{81}	Affinity of P_{OLas} for LasR regulators	6.00×10^{-3}	$[10^{-6}, 10^2]$	Log
K_{D6}	Affinity of LuxR-HSL_6 tetramerisation	3.44×10^{-2}	$[10^{-8}, 10^0]$	Log
K_{D12}	Affinity of LuxR-HSL_{12} tetramerisation	7.63×10^{-4}	$[10^{-8}, 10^0]$	Log
K_{E6}	Affinity of LasR-HSL_6 tetramerisation	3.27×10^{-5}	$[10^{-8}, 10^0]$	Log
K_{E12}	Affinity of LasR-HSL_{12} tetramerisation	3.96×10^{-2}	$[10^{-8}, 10^0]$	Log
K_{R6}	Affinity of LuxR-HSL_6 dimerisation	1.68×10^{-2}	$[10^{-8}, 10^2]$	Log
K_{R12}	Affinity of LuxR-HSL_{12} dimerisation	1.07×10^{-2}	$[10^{-8}, 10^2]$	Log
K_{S6}	Affinity of LasR-HSL_6 dimerisation	7.57×10^{-5}	$[10^{-8}, 10^2]$	Log
K_{S12}	Affinity of LasR-HSL_{12} dimerisation	1.42×10^{-1}	$[10^{-8}, 10^2]$	Log
ρ_R	LuxR scale (see Appendix B.2)	7.52×10^3	$[10^{-3}, 10^4]$	Log
ρ_S	LasR scale (see Appendix B.2)	2.92×10^3	$[10^{-3}, 10^4]$	Log
n	Stoichiometry of HSL molecules (see Appendix B.3)	1.195	$[10^{-2}, 10^5]$	Real
a_0^A	Basal transcription rate of pBad	0.877	$[0.0, 10.0]$	Real
a_1^A	Maximum transcription of pBad	5440	$[10^{-1}, 10^4]$	Real
K_A	Half-saturation constant of pBad induction by arabinose	3.707×10^3	$[10^{-5}, 10^5]$	Log
n_A	Stoichiometry of arabinose	0.99	$[0.5, 2.5]$	Real
s_{bad}	Scale factor of LasR/LuxR levels from P_{bad} (see Appendix B.4)	0.284	$[10^{-2}, 10^5]$	Log

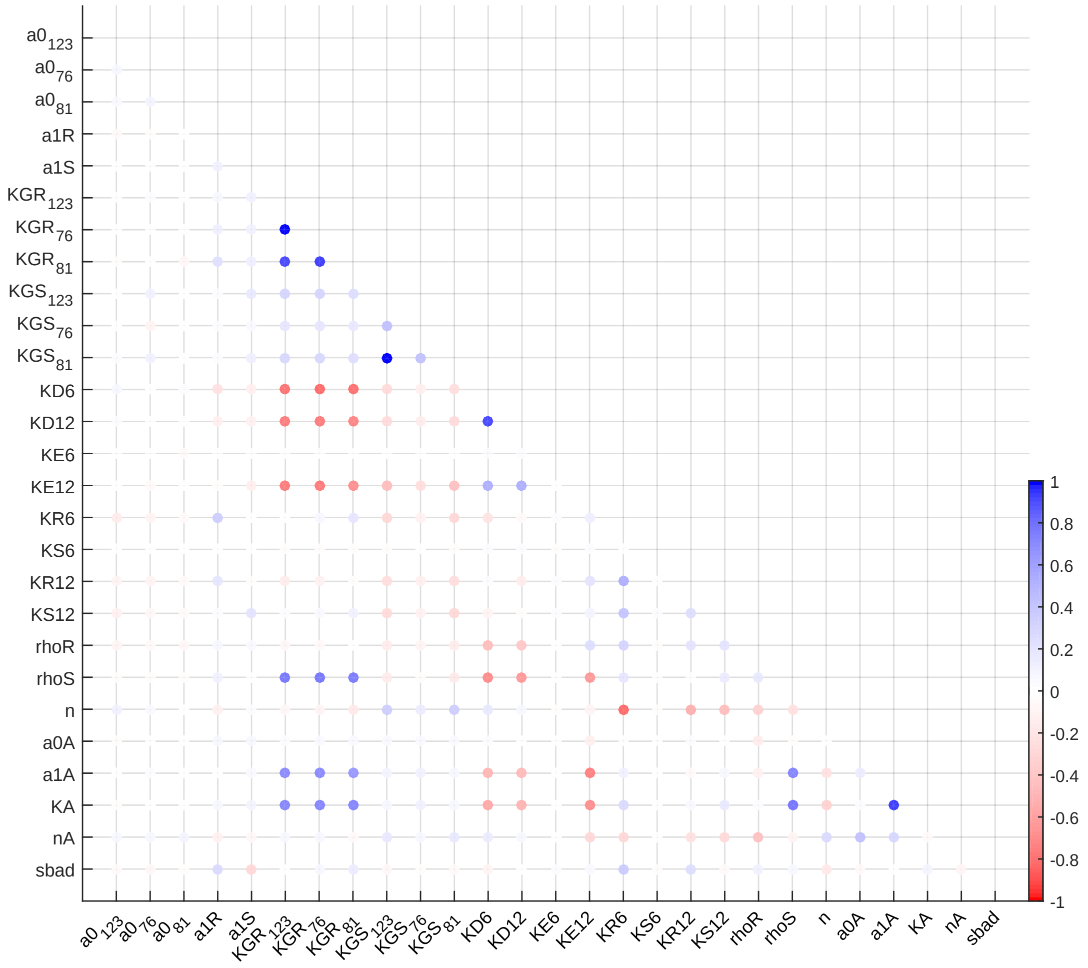

Figure S3: Parameter correlations within the joint posterior parameter distribution for the full model. The correlation between each pair of parameters was calculated using the thinned marginal posterior samples, using the `corrcoef` function in Matlab. Strong positive correlations are indicated in blue, while strong negative correlations are indicated in red.

B.6 Model-data comparison

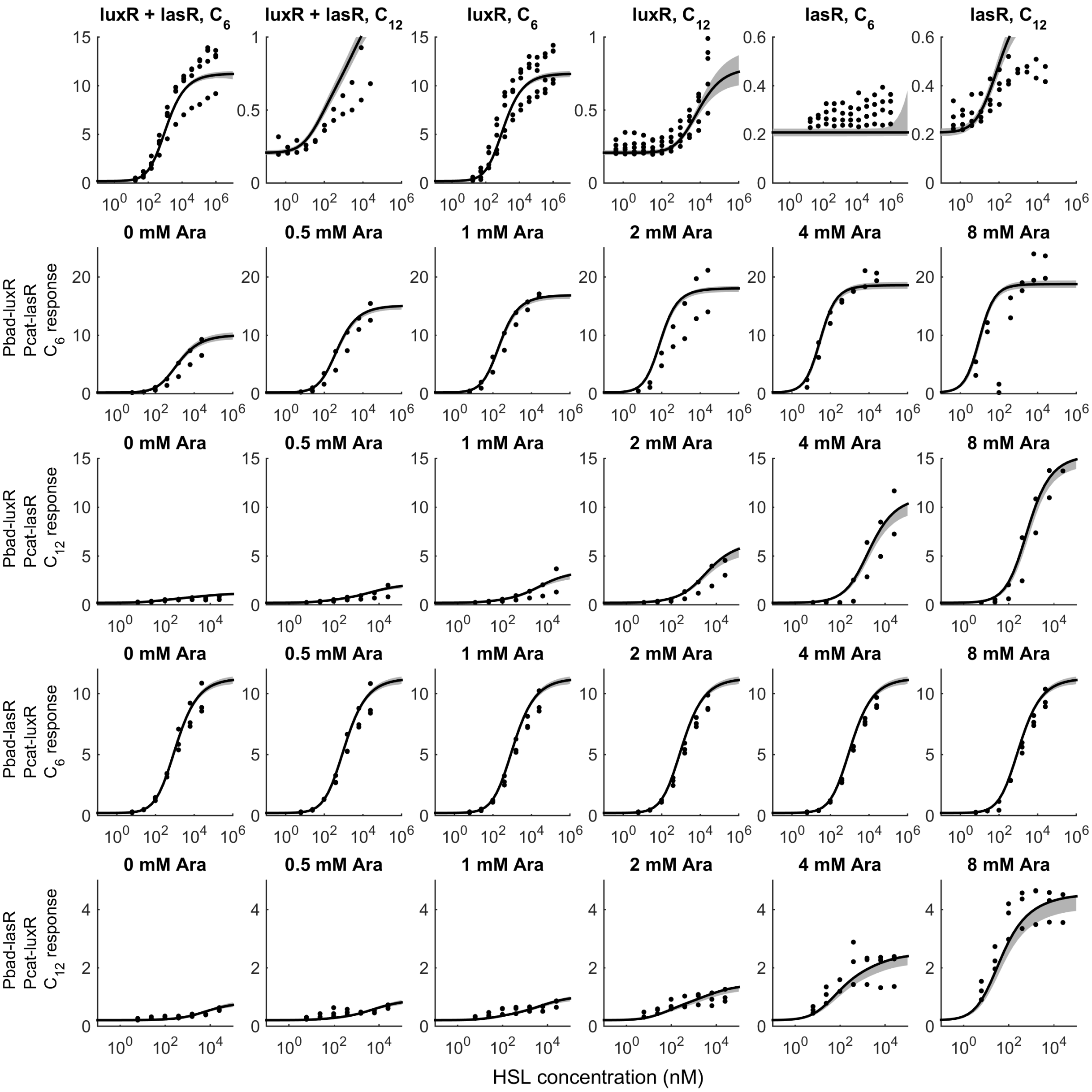

Figure S4: Model-data comparison for the wild-type pLux promoter. Experimental measurements are shown as circles, and model behavior as black lines and grey areas. Experimental measurements use the ratiometric characterization method in [2], driving YFP with P_{Lux} and normalising by measurements of CFP driven by P_{std} (J23101). Model behaviors were obtained by simulating the thinned joint posterior and calculating the mean and 95% confidence intervals. The top row has data where LuxR and LasR are expressed by pcat, while the other rows use arabinose-inducible expression of either LuxR or LasR, as specified by the left-hand labels. The concentration of arabinose is indicated above each panel.

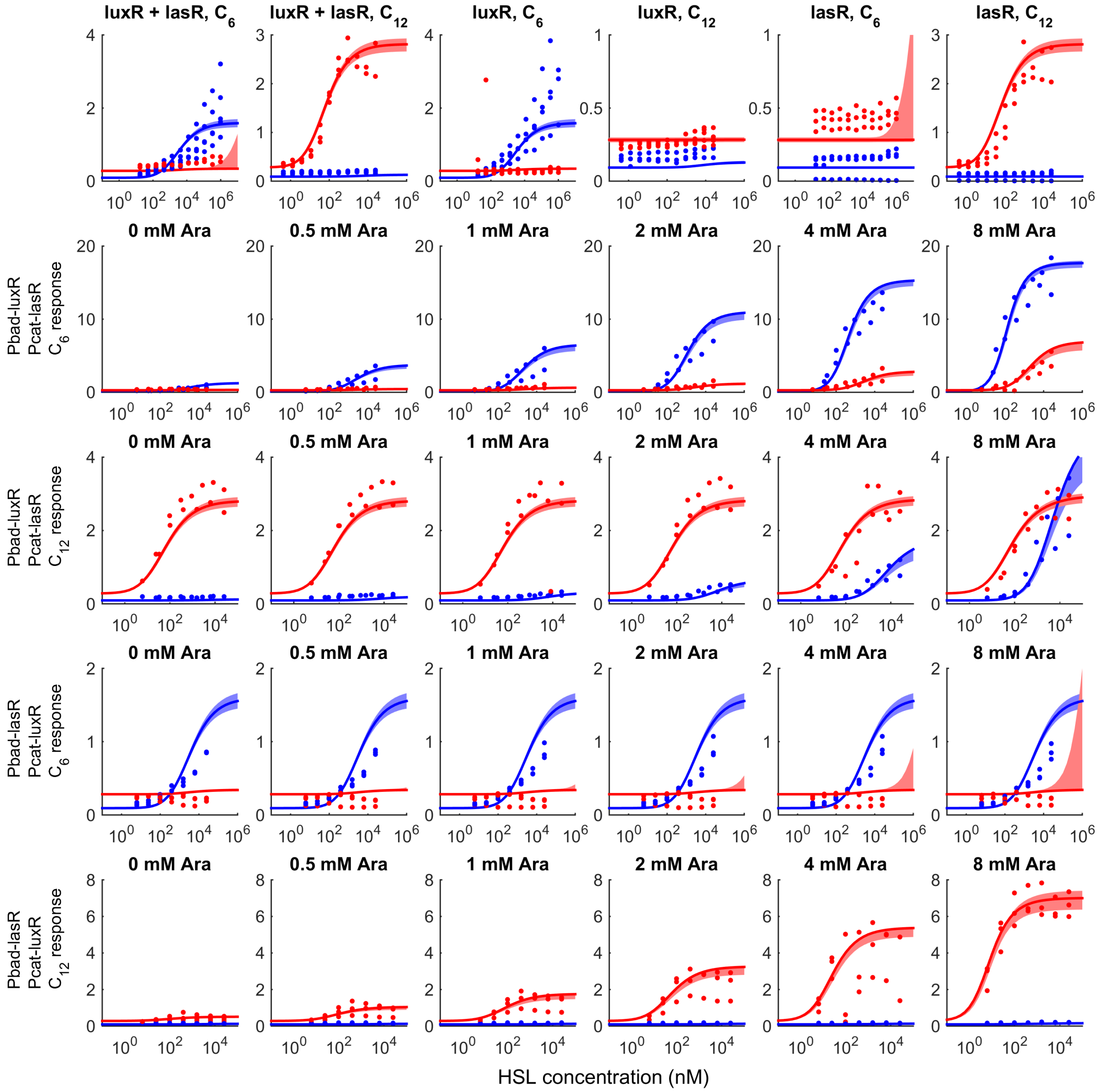

Figure S5: Model-data comparison for the mutant pLux promoters. Experimental measurements are shown as circles, and model behavior as solid lines and lighter shaded areas. Overlaid are simulations and measurements of the pLux76promoter (blue) and the pLas81promoter (red). Experimental measurements use the ratiometric characterization method in [2], driving YFP with pLux76/pLas81and normalising by measurements of CFP driven by P_{std} (J23101). Model behaviors were obtained by simulating the thinned joint posterior and calculating the mean and 95% confidence intervals. The top row has data where LuxR and LasR are expressed by pcat, while the other rows use arabinose-inducible expression of either LuxR or LasR, as specified by the left-hand labels. The concentration of arabinose is indicated above each panel.

C A simplified model that has a closed-form solution

C.1 Derivation of the transfer function

We found that a closed-form solution could be obtained when making the assumption that cell growth does not dilute the D_k and $G.D_k$ (and accordingly E_k, $G.E_k$). As such, we also remove the term for plasmid production n_G. Applying this assumption leads to the following modified version of equations (2):

$$\frac{d[R]}{dt} = n_R + u_{Rk}[R_k] - [R](\gamma + b_{Rk}[C_k]) \tag{22a}$$

$$\frac{d[R_k]}{dt} = b_{Rk}[R][C_k] + 2u_{Dk}[D_k] - [R_k](\gamma + u_{Rk} + 2b_{Dk}[R_k]) \tag{22b}$$

$$\frac{d[D_k]}{dt} = b_{Dk}[R_k]^2 + u_{GDk}[G.D_k] - [D_k](u_{Dk} + b_{GDk}[G]) \tag{22c}$$

$$\frac{d[G]}{dt} = u_{GDk}[G.D_k] - b_{GDk}[D_k][G] \tag{22d}$$

$$\frac{d[G.D_k]}{dt} = b_{GDk}[G][D_k] - u_{GDk}[G.D_k] \tag{22e}$$

Solving (22) equal to zero, we obtain the equilibria

$$[G.D_k]^* = K_{GDk}[G][D_k], \quad [D_k]^* = K_{Dk}[R_k]^2, \quad [R_k]^* = K_{Rk}[R][C_k], \quad [R]^* = \frac{r}{1 + K_{Rk}C_k} \tag{23}$$

where $K_{Rk} = \dfrac{b_{Rk}}{\gamma + u_{Rk}}$, $K_{Dk} = \dfrac{b_{Dk}}{u_{Dk}}$, $K_{GDk} = \dfrac{b_{GDk}}{u_{GDk}}$ and $r = \dfrac{n_R}{\gamma}$. Therefore (also symmetry of R and S),

$$[G.D_k]^* = K_{GDk}K_{Dk}\left(\frac{K_{Rk}[C_k]r}{1 + K_{Rk}[C_k]}\right)^2 \tag{24a}$$

$$[G.E_k]^* = K_{GEk}K_{Ek}\left(\frac{K_{Sk}[C_k]s}{1 + K_{Sk}[C_k]}\right)^2 \tag{24b}$$

where the new K's are defined as above, and $s = \dfrac{n_S}{\gamma}$.

By taking advantage of the conservation law $[G] + [G.D_6] + [G.D_{12}] + [G.E_6] + [G.E_{12}] = \text{constant}$, we can derive the rate of production of mRNA as a function of r, s and C_k as

$$\frac{a_0 + a_{Rk}K_{GRk}\left(\dfrac{K_{Rk}C_k r}{1 + K_{Rk}C_k}\right)^2 + a_{Sk}K_{GSk}\left(\dfrac{K_{Sk}C_k s}{1 + K_{Sk}C_k}\right)^2}{1 + K_{GRk}\left(\dfrac{K_{Rk}C_k r}{1 + K_{Rk}C_k}\right)^2 + K_{GSk}\left(\dfrac{K_{Sk}C_k s}{1 + K_{Sk}C_k}\right)^2} \tag{25}$$

where $K_{GRk} = K_{GDk}K_{Dk}$ and $K_{GSk} = K_{GEk}K_{Ek}$.

As before, we replaced the exponent 2 of the HSL concentration with a parameter to-be-inferred, giving

$$\frac{a_0 + a_{Rk}K_{GRk}r^2\left(\dfrac{K_{Rk}C_k}{1 + K_{Rk}C_k}\right)^n + a_{Sk}K_{GSk}s^2\left(\dfrac{K_{Sk}C_k}{1 + K_{Sk}C_k}\right)^n}{1 + K_{GRk}r^2\left(\dfrac{K_{Rk}C_k}{1 + K_{Rk}C_k}\right)^n + K_{GSk}s^2\left(\dfrac{K_{Sk}C_k}{1 + K_{Sk}C_k}\right)^n} \tag{26}$$

C.2 The functional response to combinations of C_6 and C_{12}.

In order to associate a single response function with our double receiver, we sought a transfer function of both inputs, HSL_6 and HSL_{12}. To achieve this, we combined equations (22)b–e for the $k = 6$ and $k = 12$ and extended the expression for free LuxR (Eqn. (22)a). We then added the equivalent equations for LasR, as in the previous sections. By solving the combined system at equilibrium, making mostly the same simplifications/substitutions as before but additionally $a_{R6} = a_{R12} = a_1^R$ and $K_{GR6} = K_{GR12} = K_{GR}$, we obtained the expression

$$\frac{a_0 + a_1^R K_{GR} r^2 \dfrac{K_{R6}^n C_6^n + K_{R12}^n C_{12}^n}{(1 + K_{R6}C_6 + K_{R12}C_{12})^n} + a_1^S K_{GS} s^2 \dfrac{K_{S6}^n C_6^n + K_{S12}^n C_{12}^n}{(1 + K_{S6}C_6 + K_{S12}C_{12})^n}}{1 + K_{GR} r^2 \dfrac{K_{R6}^n C_6^n + K_{R12}^n C_{12}^n}{(1 + K_{R6}C_6 + K_{R12}C_{12})^n} + K_{GS} s^2 \dfrac{K_{S6}^n C_6^n + K_{S12}^n C_{12}^n}{(1 + K_{S6}C_6 + K_{S12}C_{12})^n}} \tag{27}$$

C.3 Parameter inference

The simplified model has 6 fewer parameters than the full model. The parameter values were inferred using Filzbach, as before. The overall model performance was numerically weaker than the full model, with the maximum likelihood being ≈ -615.5. However, all marginal posterior distributions were more convincingly Gaussian-shaped (Fig. S6) than in the full model. The only parameters that were not possible to identify were the maximum transcription rate and half-saturation constants relating to the pBad promoter. This was not surprising, as we did not explore the upper end of the induction curve for the pBad promoter, because we found that arabinose concentrations beyond 10 mM had adverse growth effects on the cells.

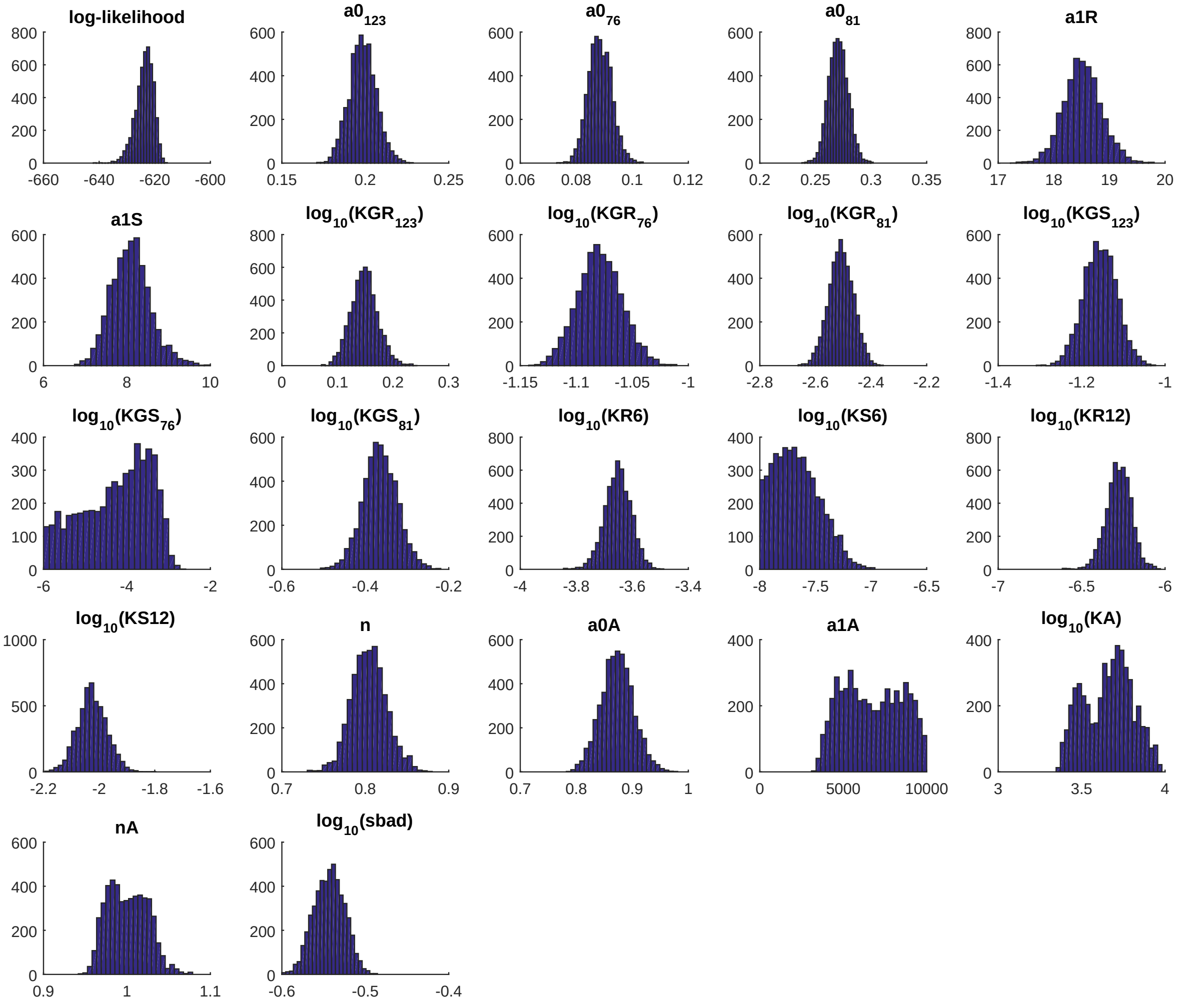

Figure S6: Marginal posterior parameter distributions for the simplified model. The marginal parameter posterior distributions are shown for each inferred parameter in the simplified model. Parameters for which the base-10 logarithm is shown are those parameters which used a logarithmic proposal distribution, while other parameters had real-valued proposals. The histograms shown represent every 50th sample out of a total of 250,000 samples in a single MCMC chain. The maximum log-likelihood score attained was -623.7.

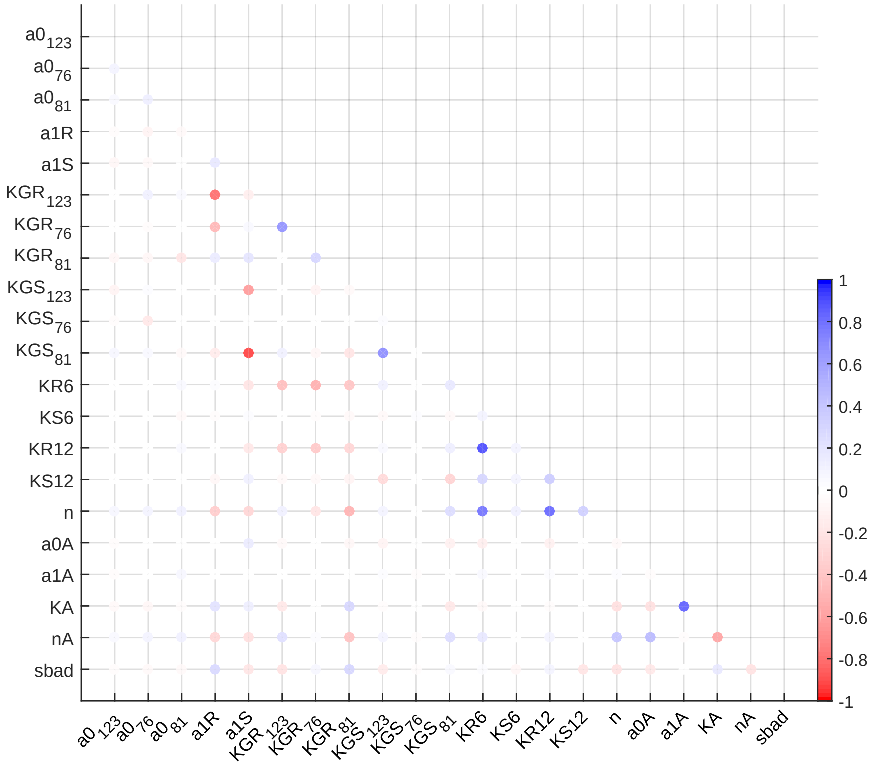

Figure S7: Parameter correlations within the joint posterior parameter distribution for the simplified model. The correlation between each pair of parameters was calculated using the thinned marginal posterior samples, using the `corrcoef` function in Matlab. Strong positive correlations are indicated in blue, while strong negative correlations are indicated in red.

Table S6: Parameters Summary. Each parameter of the full model is defined, along with its maximum likelihood estimate (MLE). For parameter inference, all prior distributions were uniform, with the ranges specified below. The proposal distributions for some parameters were set to a logarithmic scale, as indicated below.

Parameter	Description	MLE	Prior	Scale
a_0^{123}	Basal transcription rate of pLux	0.199	$[0, 10]$	Real
a_0^{76}	Basal transcription rate of pLux76	0.086	$[0, 10]$	Real
a_0^{81}	Basal transcription rate of pLas81	0.264	$[0, 10]$	Real
a_1^{R}	Transcription rate with LuxR regulators bound	18.47	$[0, 100]$	Real
a_1^{S}	Transcription rate with LasR regulators bound	8.24	$[0, 100]$	Real
K_{GR}^{123}	P_{Lux} binding and tetramizeration for LuxR regulators	1.419×10^{0}	$[10^{-6}, 10^{2}]$	Log
K_{GR}^{76}	P_{OLux} binding and tetramizeration for LuxR regulators	8.657×10^{-2}	$[10^{-6}, 10^{2}]$	Log
K_{GR}^{81}	P_{OLas} binding and tetramizeration for LuxR regulators	3.329×10^{-3}	$[10^{-6}, 10^{2}]$	Log
K_{GS}^{123}	P_{Lux} binding and tetramizeration for LasR regulators	7.186×10^{-2}	$[10^{-6}, 10^{2}]$	Log
K_{GS}^{76}	P_{OLux} binding and tetramizeration for LasR regulators	4.788×10^{-4}	$[10^{-6}, 10^{2}]$	Log
K_{GS}^{81}	P_{OLas} binding and tetramizeration for LasR regulators	4.249×10^{-1}	$[10^{-6}, 10^{2}]$	Log
K_{R6}	Affinity of LuxR-HSL_6 dimerization	2.076×10^{-4}	$[10^{-8}, 10^{2}]$	Log
K_{R12}	Affinity of LuxR-HSL_{12} dimerization	4.937×10^{-7}	$[10^{-8}, 10^{2}]$	Log
K_{S6}	Affinity of LasR-HSL_6 dimerization	1.710×10^{-8}	$[10^{-8}, 10^{2}]$	Log
K_{S12}	Affinity of LasR-HSL_{12} dimerization	8.827×10^{-3}	$[10^{-8}, 10^{2}]$	Log
n	Stoichiometry of HSL molecules (see Appendix B.3)	0.797	$[0.5, 2.5]$	Real
a_0^{A}	Basal transcription rate of pBad	0.874	$[0.0, 10.0]$	Real
a_1^{A}	Maximum transcription of pBad	8864	$[10^{-1}, 10^{4}]$	Real
K_A	Half-saturation constant of pBad induction by arabinose	6.750×10^{3}	$[10^{-5}, 10^{5}]$	Log
n_A	Stoichiometry of arabinose	0.99	$[0.5, 2.5]$	Real
s_{bad}	Scale factor of LasR/LuxR levels from P_{bad} (see Appendix B.4)	0.282	$[10^{-2}, 10^{5}]$	Log

D Characterization using GEC

In previous work [2], computational procedures for ratiometric characterization were implemented as part of the Visual GEC software framework [7]. While the focus there was on the characterization of a single device, in this work the simultaneous characterization of multiple devices using a number of different data sets is required. These devices share a number of parts and, subsequently, the computational models describing their behaviors have shared parameters. In this section, we describe the main extensions to the GEC language and relevant methods that were implemented as a prototype within Visual GEC in order to support the definition of (i) modules, (ii) shared parameters and (iii) module response functions, allowing the results of this study to be reproduced directly in the GEC framework.

To allow the specification of multiple devices within GEC, we exploited the concept of *modules* introduced in [7]. However, for the purpose of characterization, the composition of modules as part of a GEC program was treated differently than in [7], where each module was characterized in isolation. Furthermore, we extended GEC to allow the definition of a set of parameters shared between different modules, together with a relevant range of values for each parameter to be considered during characterization.

To allow the specification of arbitrary models describing the input/output behavior of modules, we extended the GEC language to support the annotation of modules with *response* functions. The responses of simple devices such as single inducible promoters, could be described by a pre-defined response function with unknown parameters (e.g. based on the Hill or Michaelis-Menten models), which was the approached followed in [2]. To handle more general models, we extended the GEC language to support the definition of response functions as symbolic expressions constructed using basic arithmetic operations (addition, subtraction, multiplication, division, exponentiation). The terms of these response functions represent constants, quantities that are measured experimentally and will be provided as part of the data sets used for characterization (e.g. the concentrations of various inducers), or the unknown, pre-defined parameters that must be characterized.

As part of the study presented in this paper, the extensions to GEC described above allowed us to represent the devices that were considered for characterization (Table S4), the (shared) model parameters from Table S6 and the response functions for each device derived in Appendix C (Program S1). A prototype command-line implementation (available from http://biology.azurewebsites.net/gec/beta/LuxLasGEC.zip) processed the experimental data to compute the relative steady-state expression observed in each condition, and then constrained all parameters of the response functions simultaneously. Currently, this prototype has limited visualization functionality and, therefore, Matlab was used to visualize the resulting parameter posteriors and models. The models, which were encoded as the response functions defined as part of the GEC program, were exported automatically as Matlab functions.

The concept of GEC modules, together with the support of of parameterized modules, allowed a concise definition of the devices considered in this study. Furthermore, the annotation of GEC modules with response functions provided a convenient strategy for formalizing the structure of devices (as in [7]) together with their hypothesized behavior. However, at present our method does not implement methods to assure that the response function proposed for a module describes its behavior adequately and response functions are treated as assumptions encoded directly by the user.

Program S1: GEC program for characterization of pLux promoters

```
directive parameters [
    a0,        (0.0, 10.0),  0.14,  real,  random
  ; a1R,       (0.0, 100.0), 10.0,  real,  random
  ; a1S,       (0.0, 100.0), 10.0,  real,  random
  ; KGR_123,   (1e-6, 1e2),  1e-2,  log,   random
  ; KGS_123,   (1e-6, 1e2),  1e-2,  log,   random
  ; KGR_76,    (1e-6, 1e2),  1e-2,  log,   random
  ; KGS_76,    (1e-6, 1e2),  1e-2,  log,   random
  ; KGR_81,    (1e-6, 1e2),  1e-2,  log,   random
  ; KGS_81,    (1e-6, 1e2),  1e-2,  log,   random
  ; KR6,       (1e-10, 1e2), 1e-2,  log,   random
  ; KS6,       (1e-10, 1e2), 1e-6,  log,   random
  ; KR12,      (1e-10, 1e2), 1e-8,  log,   random
  ; KS12,      (1e-10, 1e2), 1e-2,  log,   random
  ; n,         (0.5, 2.5),   2.0,   real,  random
  ; Aa0,       (0.0, 10.0),  0.0,   real,  random
  ; Aa1,       (0.0, 300.0), 10.0,  real,  random
  ; AK,        (1e-12, 1e5), 1.0,   log,   random
  ; An,        (0.0, 10.0),  1.0,   real,  random
  ; sbad,      (1e-2, 1e5),  1.0,   log,   random]

module PcatLL(KGR, KGS, pLux) {
    response {
        PoPS = ((a0 + a1R*KGR*(1.0**2.0)*(((KR6**n)*(C6**n) + (KR12**n)*(C12**n))/((1.0 + KR6*C6 +
            KR12*C12)**n)) + a1S*KGS*(1.0**2.0)*(((KS6**n)*(C6**n) + (KS12**n)*(C12**n))/((1.0 +
            KS6*C6 + KS12*C12)**n)))/(1.0 + KGR*(1.0**2.0)*(((KR6**n)*(C6**n) +
            (KR12**n)*(C12**n))/((1.0 + KR6*C6 + KR12*C12)**n)) + KGS*(1.0**2.0)*(((KS6**n)*(C6**n)
            + (KS12**n)*(C12**n))/((1.0 + KS6*C6 + KS12*C12)**n)))) }
    pCat:prom; b0034:rbs; luxR:pcr; b0034:rbs; lasR:pcr; ter; pLux:prom; b0034:rbs; eYFP:pcr; ter;
        pCI:prom; b0034:rbs; eCFP:pcr; ter };

module PcatR(KGR, pLux) {
    response {
        PoPS = ((a0 + a1R*KGR*(1.0**2.0)*(((KR6**n)*(C6**n) + (KR12**n)*(C12**n))/((1.0 + KR6*C6 +
            KR12*C12)**n)) )/(1.0 + KGR*(1.0**2.0)*(((KR6**n)*(C6**n) + (KR12**n)*(C12**n))/((1.0 +
            KR6*C6 + KR12*C12)**n)))) }
    pCat:prom; b0034:rbs; luxR:pcr; ter; pLux:prom; b0034:rbs; eYFP:pcr; ter; pCI:prom; b0034:rbs;
        eCFP:pcr; ter };

module PcatS(KGS, pLux) {
    response {
        PoPS = ((a0 + a1S*KGS*(1.0**2.0)*(((KS6**n)*(C6**n) + (KS12**n)*(C12**n))/((1.0 + KS6*C6 +
            KS12*C12)**n)) )/(1.0 + KGS*(1.0**2.0)*(((KS6**n)*(C6**n) + (KS12**n)*(C12**n))/((1.0 +
            KS6*C6 + KS12*C12)**n)))) }
    pCat:prom; b0034:rbs; lasR:pcr; ter; pLux:prom; b0034:rbs; eYFP:pcr; ter; pCI:prom; b0034:rbs;
        eCFP:pcr; ter };

module PbadR(KGR, KGS, pLux) {
    response {
        PoPS = ((a0 + a1R*KGR*(1.0**2.0)*(((KR6**n)*(C6**n) + (KR12**n)*(C12**n))/((1.0 + KR6*C6 +
            KR12*C12)**n)) + a1S*KGS*((sbad * ((Aa0*AK**An + Aa1*Ara**An)/(AK**An +
            Ara**An)))**2.0)*(((KS6**n)*(C6**n) + (KS12**n)*(C12**n))/((1.0 + KS6*C6 +
            KS12*C12)**n)))/(1.0 + KGR*(1.0**2.0)*(((KR6**n)*(C6**n) + (KR12**n)*(C12**n))/((1.0 +
```

```
            KR6*C6 + KR12*C12)**n)) + KGS*((sbad * ((Aa0*AK**An + Aa1*Ara**An)/(AK**An +
            Ara**An)))**2.0)*(((KS6**n)*(C6**n) + (KS12**n)*(C12**n))/((1.0 + KS6*C6 +
            KS12*C12)**n)))) }
    pCat:prom; b0034:rbs; luxR:pcr; ter; pLux:prom; b0034:rbs; eYFP:pcr; ter; pCI:prom; b0034:rbs;
        eCFP:pcr; ter |
    pBad:prom; b0034:rbs; lasR:pcr; ter };

module PbadS(KGR, KGS, pLux) {
    response {
        PoPS = ((a0 + a1R*KGR*(((Aa0*AK**An + Aa1*Ara**An)/(AK**An +
            Ara**An))**2.0)*(((KR6**n)*(C6**n) + (KR12**n)*(C12**n))/((1.0 + KR6*C6 +
            KR12*C12)**n)) + a1S*KGS*(1.0**2.0)*(((KS6**n)*(C6**n) + (KS12**n)*(C12**n))/((1.0 +
            KS6*C6 + KS12*C12)**n)))/(1.0 + KGR*(((Aa0*AK**An + Aa1*Ara**An)/(AK**An +
            Ara**An))**2.0)*(((KR6**n)*(C6**n) + (KR12**n)*(C12**n))/((1.0 + KR6*C6 +
            KR12*C12)**n))+ KGS*(1.0**2.0)*(((KS6**n)*(C6**n) + (KS12**n)*(C12**n))/((1.0 + KS6*C6
            + KS12*C12)**n)))) }
    pCat:prom; b0034:rbs; lasR:pcr; ter; pLux:prom; b0034:rbs; eYFP:pcr; ter; pCI:prom; b0034:rbs;
        eCFP:pcr; ter |
    pBad:prom; b0034:rbs; luxR:pcr; ter };

(* Wild type Pcat devices *)
PcatLL{Pcat_LL123}(KGR_123, KGS_123, pLux) |
PcatR{Pcat_R123}(KGR_123, pLux) |
PcatS{Pcat_S123}(KGS_123, pLux) |

(* Wild type Pbad devices *)
PbadR{Pbad_LasR_R123}(KGR_123, KGS_123, pLux) |
PbadS{Pbad_LuxR_S123}(KGR_123, KGS_123, pLux) |

(* 76 mutant Pcat devices *)
PcatLL{Pcat_LL76}(KGR_76, KGS_76, pLux76) |
PcatR{Pcat_R76}(KGR_76, pLux76) |
PcatS{Pcat_S76}(KGS_76, pLux76) |

(* 81 mutant Pcat devices *)
PcatLL{Pcat_LL81}(KGR_81, KGS_81, pLas81) |
PcatR{Pcat_R81}(KGR_81, pLas81) |
PcatS{Pcat_S81}(KGS_81, pLas81) |

(* 76 mutant Pbad devices *)
PbadR{Pbad_LasR_R76}(KGR_76, KGS_76, pLux76) |
PbadS{Pbad_LuxR_S76}(KGR_76, KGS_76, pLux76) |

(* 81 mutant Pbad devices *)
PbadR{Pbad_LasR_R81}(KGR_81, KGS_81, pLas81) |
PbadS{Pbad_LuxR_S81}(KGR_81, KGS_81, pLas81)
```

E Reducing crosstalk

E.1 Model-based determination of optimal LuxR and LasR levels

To determine the optimal LuxR and LasR levels to be used within the same cell, we used the parameterized model to calculate a metric analogous to the signal-noise ratio for each signalling channel. Specifically, we considered the promoter activity at $\approx 1\mu$M of HSL_6 or HSL_{12}, and varied the LuxR and LasR levels. We then sought to maximize the ratio of HSL_6 response to HSL_{12}12 response in pLux76and the opposite in pLas81. i.e.

$$\max_{R,S} \left\{ \frac{f_6^{P76}(R,S)}{f_{12}^{P76}(R,S)} \cdot \frac{f_{12}^{P81}(R,S)}{f_6^{P81}(R,S)} \right\} \tag{28}$$

This choice of cost function produced a convex optimization problem that was easy to solve numerically. In other words, there was a clear peak combination of LuxR and LasR levels.

We can generate different responses with different combinations of (LuxR,LasR). Here we've optimized for the ratio of expression, while we might well have optimized for minimal crosstalk. However, we would expect that this comes at the cost of absolute expression.

E.2 Characterization of double receivers

Double receiver cells were created and characterized as described in the main text (also summarized in Figure 3). To force the LuxR and LasR levels closer to the model-suggested optimum (Figure 3B), the ribosome binding sites associated with LuxR and LasR translation were modified (Figure 3A). Several different RBSes were used, and their predicted efficiencies were obtained from the RBS calculator [3] (Table S7).

Table S7: Predicted and inferred translation of LuxR and LasR for different ribosome binding sites. Compared are LuxR/LasR levels inferred from the modeling process with translation efficiency predicted by the RBS calculator [3]. The modeling calibrated LuxR and LasR levels with the double receiver data shown in Figure S8.

Background	LuxR100	LuxR33	LasR32	LasR34	LasR175	$\frac{\text{LuxR33}}{\text{LuxR100}}$	$\frac{\text{LasR34}}{\text{LasR32}}$	$\frac{\text{LasR175}}{\text{LasR32}}$
pR100S32	1.3584		0.6409					
pR100S34	0.9450			10.1890				
pR33S32		5.5539	0.6583					
pR33S34		4.1562		10.8429				
pR33S175		5.8881			2.9688			
Inferred mean	1.1517	5.1994	0.6496	10.5160	2.9688	4.8236	22.8166	6.1205
RBS calculator	372.49	96.78	37.82	518.14	168.2	3.8488	13.7002	4.4474

The transfer functions for 5 combinations of modified LuxR and LasR levels are shown in Figure S8. These data were used to infer the relative LuxR and LasR levels in each device. Note that the units of relative promoter activity for the double receivers was either YFP/RFP or CFP/RFP, which meant that they needed to be converted so as to be comparable with the relative promoter activity units of YFP/CFP used in Figures 1 and 2. As such, a CFP/RFP conversion factor was determined from measurements of YFP and CFP in cells transformed with chromosomally integrated mRFP1 and pCatR34S34. The relative LuxR and LasR levels inferred using the re-calibrated data are quantified in Table S7.

Constructs in which LasR translation was regulated by RBS Bba_B0034 (pR100S34, pR33S34) displayed a transfer function whose shape was not well fit by the model. The presence of a peak at lower concentrations of 3OC12-HSL, dipping to a lower plateau at higher concentrations suggests a regulatory mechanism for which we are not accounting in our model that is revealed only when LasR is expressed at very high levels.

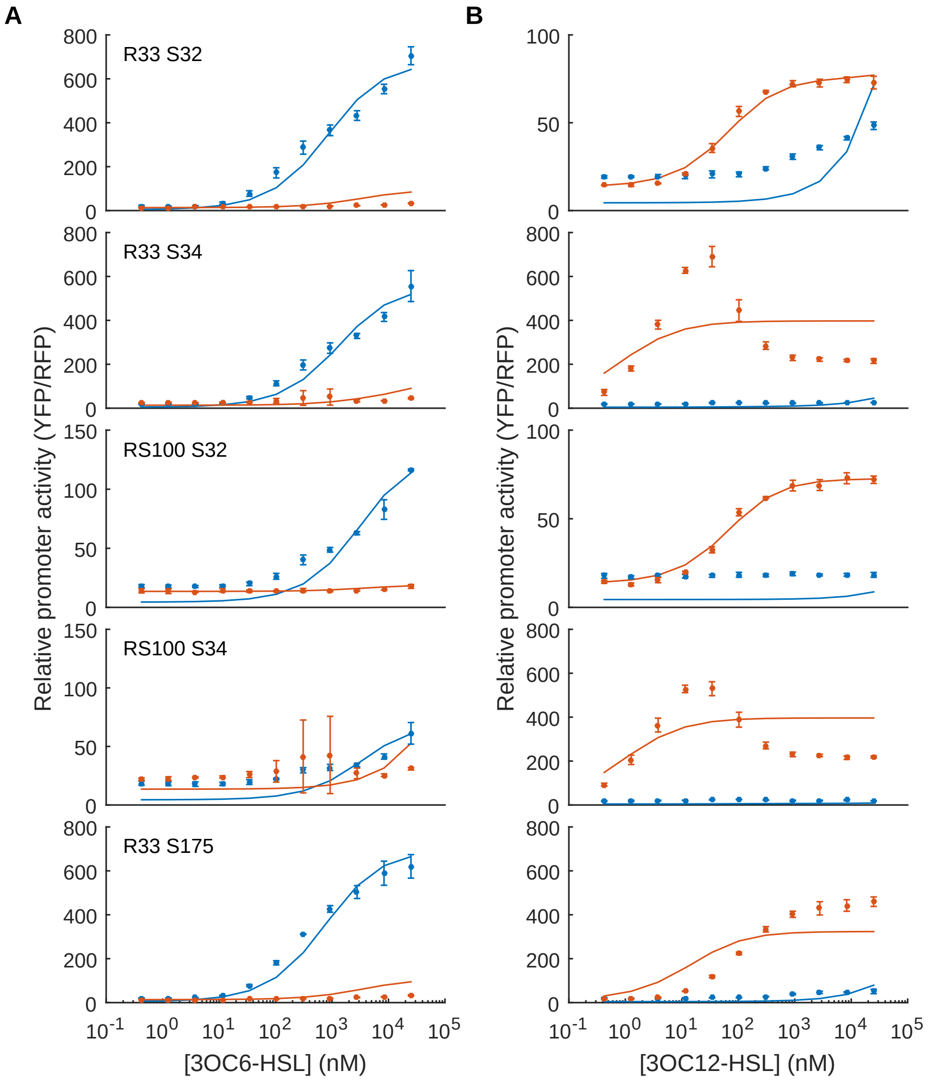

Figure S8: Characterization of double receivers. Activity (relative to chromosomal constitutive mRFP1) of pLux76 (eCFP, blue) and pLas81 (eYFP, red) in the double receiver variants as a function of 3OC6-HSL (A) or 3OC12-HSL (B).

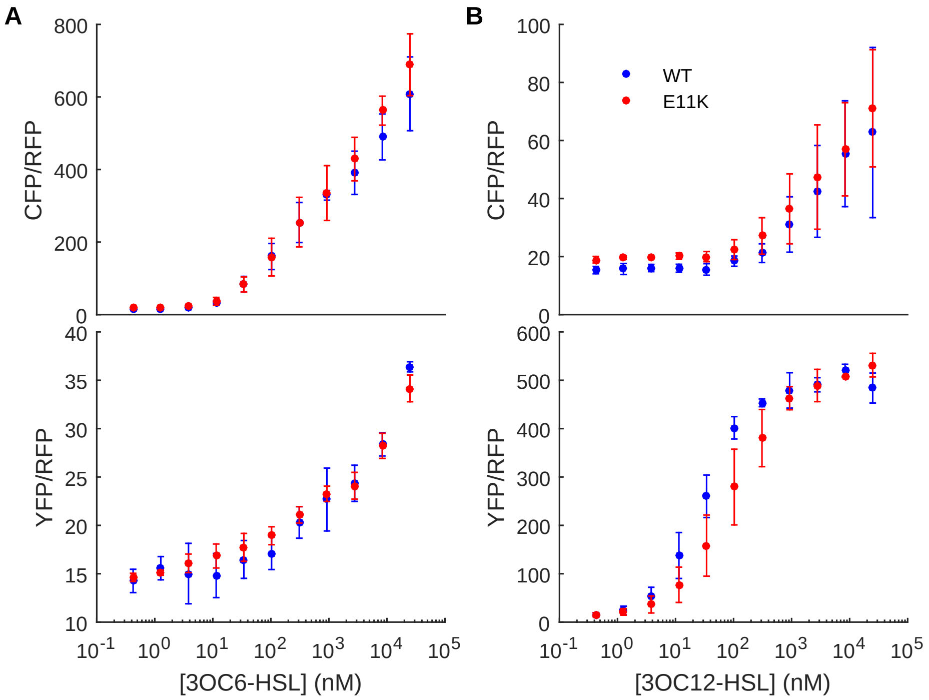

Figure S9: Comparison of LasR variants. Activity (relative to chromosomal constitutive mRFP1) of pLux76 (CFP/RFP) and pLas81 (YFP/RFP) in response to increasing concentrations of 3OC6-HSL (A) or 3OC12-HSL (B) was measured for construct pR33S175 containing either a LasR variant with the E11K substitution (red points) or WT LasR (blue points).

F Modelling spatio-temporal dynamics resulting from HSL diffusion

F.1 Characterization of HSL diffusion and sender cells

To characterize the diffusion and detection of HSLs in receiver cells, we formulated a spatial model for comparison with the experiments in Fig. 3. Cell growth is modelled using the Gompertz function [8]. We then assume that the rate of fluorescent protein synthesis is proportional to the cell density at a given location and the promoter activity of each promoter in response to each HSL molecule. Based on the 1-dimensional reaction-diffusion equations, this becomes

$$[N_R](t) = Ke^{\log(\frac{c_0}{K})e^{-rt}} \tag{29a}$$

$$\frac{\partial[C_6]}{\partial t} = -\gamma_{\text{HSL}}[C_6] + D_6\frac{\partial^2[C_6]}{\partial x^2} \tag{29b}$$

$$\frac{\partial[C_{12}]}{\partial t} = -\gamma_{\text{HSL}}[C_{12}] + D_{12}\frac{\partial^2[C_{12}]}{\partial x^2} \tag{29c}$$

$$\frac{\partial[\text{iCFP}]}{\partial t} = \alpha_C[N].f_{76}(R, S, [C_6], [C_{12}]) - (\gamma_{\text{FP}} + \mu)[\text{iCFP}] \tag{29d}$$

$$\frac{\partial[\text{iYFP}]}{\partial t} = \alpha_Y[N].f_{81}(R, S, [C_6], [C_{12}]) - (\gamma_{\text{FP}} + \mu)[\text{iYFP}] \tag{29e}$$

$$\frac{\partial[\text{iCFP}]}{\partial t} = \mu[\text{iCFP}] - \gamma_{\text{FP}}[\text{CFP}] \tag{29f}$$

$$\frac{\partial[\text{iYFP}]}{\partial t} = \mu[\text{iYFP}] - \gamma_{\text{FP}}[\text{YFP}] \tag{29g}$$

where N_R is the density of receiver cells, and iCFP/iYFP are the immature forms of CFP/YFP. D_6 and D_{12} are the HSL diffusion rates, f_{76} and f_{81} are the promoter responses to HSL molecules, as derived previously, μ is the rate of fluorescent protein maturation and γ_{FP} is the fluorescent protein degradation rate. This simple model implicitly incorporates the following assumptions:

- The mRNA dynamics are faster than the protein dynamics, allowing their contribution to be incorporated into the synthesis of fluorescent protein
- Cells are immotile

To describe HSL sender cells at each end of the domain, we assume that the cells synthesize HSL at a constant rate, so bulk synthesis is proportional to the density of each sender cell type. i.e.

$$[N_{S6}](t) = Ke^{\log(\frac{c_0}{K})e^{-rt}} \tag{30a}$$

$$[N_{S12}](t) = Ke^{\log(\frac{c_0}{K})e^{-rt}} \tag{30b}$$

$$\frac{\partial[C_6]}{\partial t} = N_{S6}.\alpha_6 - \gamma_{\text{HSL}}[C_6] + D_6\frac{\partial^2[C_6]}{\partial x^2} \tag{30c}$$

$$\frac{\partial[C_{12}]}{\partial t} = N_{S12}.\alpha_{12} - \gamma_{\text{HSL}}[C_{12}] + D_{12}\frac{\partial^2[C_{12}]}{\partial x^2} \tag{30d}$$

(30e)

where α_6 and α_{12} are the per-capita rates of HSL production in each sender cell. The initial conditions of the simulation are then imposed to correspond to the experimental setup, specifying an initial density of each cell type in the relevant locations. Finally, we note that all spatial simulations in this article used zero-flux boundary conditions.

Inferring cell growth parameters

Since f_{76} and f_{81} had already been parameterized from steady-state measurements of fluorescence protein production (Figs. 1 & 2), the remaining unidentified parameters in the system were r, γ_{HSL}, D_6, D_{12}, γ_{FP}, α_6 and α_{12}. We fitted r and γ_{FP} separately, since they could be obtained purely from the RFP channel of the dataset. We approximated r using the measured RFP traces in the plate of Fig. 3, fitting the parameters of the growth model. Briefly, the model is given by

$$\frac{d[\text{RFP}]}{dt} = c(t; r, 1, c_0) - \gamma_{\text{RFP}}[\text{RFP}] \tag{31}$$

where $c(t)$ is the solution to the growth model being used, with growth rate r, carrying capacity assumed equal to 1, and initial cell density c_0. To relate the simulation to the fluorescence data, an optimal scale factor λ was selected, and a cost function defined as

$$\Delta_R = \sum_k \left(y_R(t_k) - \lambda.[\text{RFP}](t_k)\right)^2 \tag{32}$$

where $y_R(t_k)$ is the mean measured RFP signal at time $t = t_k$. The value of λ was evaluated using standard linear regression techniques (with a zero intercept). The then used the `fminsearch` function in Matlab to find solutions to the minimization problem

$$\min_{r, c_0, \gamma_{\text{FP}}} \Delta_R \tag{33}$$

We obtained a very close fit to the data (Fig. S10; see Table S8 for parameter values).

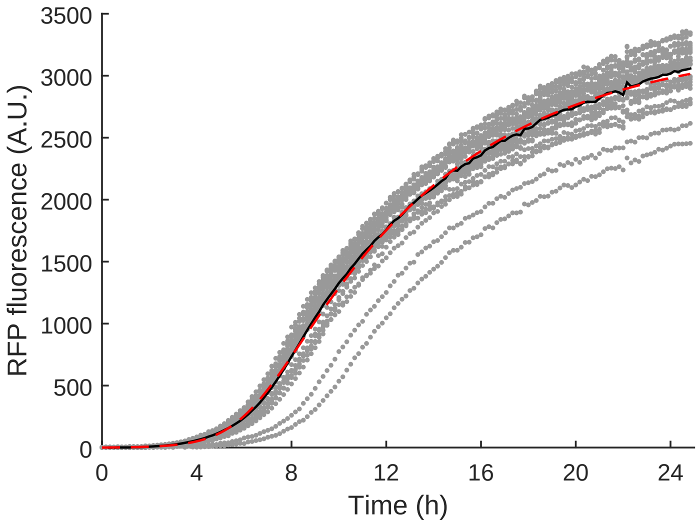

Figure S10: Fitting the Gompertz growth parameters to RFP data. The Gompertz growth parameters were inferred using the `fminsearch` function in Matlab. Shown are the RFP measurements from each cell colony (grey dots), their average at each time-point (solid black line), and the model simulation of best fit parameters (dashed red line). See Table S8 for parameter values.

Inferring HSL parameters

The remaining uncharacterized parameters were inferred from the data in Fig. 3 of the main text. We used a similar approach to that taken above, using `fminsearch` to minimize a cost function with a scale parameter λ that is calculated using linear regression. The cost function combined data for each of the 5 double

receiver cell types shown in Fig. 3 of the main text. Additionally, each fluorescent signal was normalized by dividing the maximum fluorescence observed over the entire experiment, maintaining relative differences in fluorescence between each double receiver cell type, but removing differences in the absolute scales of CFP and YFP. Therefore, we can write the cost function as

$$\Delta_{CY} = \sum_d \sum_k (y_C(t_k) - \lambda.[\text{CFP}](t_k))^2 + (y_Y(t_k) - \lambda.[\text{YFP}](t_k))^2 \tag{34}$$

where $d \in \{pR33S34, pR33S175, pR33S32, pR100S34, pR100S32\}$ (see Table S1 for details).

The estimate of γ_{FP} for the RFP data was used as an approximation of γ_{FP} for the CFP and YFP variables in (29), reducing the number of parameters to be inferred. The remaining parameters were γ_{HSL}, D_6, D_{12}, α_6 and α_{12}. These were fit simultaneously using `fminsearch` against the CFP and YFP data. A comparison of the data against simulation of the best fit parameters is shown in Fig. 3, and uses the parameters values summarized in Table S8.

Table S8: Summary of optimized parameters for the spatial model of double receivers and HSL diffusion.

Parameter	Description	Value (Figure 3E)	Value (Figure S11)
r	Cellular growth rate	6.50×10^{-5} s^{-1}	6.22×10^{-5} s^{-1}
c_0	Initial cell density	4.72×10^{-4}	3.99×10^{-4}
γ_{FP}	Turnover of fluorescenct protein	8.38×10^{-5} s^{-1}	
μ	Maturation of CFP/YFP	2.80×10^{-4} s^{-1}	8.60×10^{-5} s^{-1}
γ_{HSL}	Turnover of HSL	1.63×10^{-4} s^{-1}	3.32×10^{-5} s^{-1}
α_6	Production of 3OC6-HSL in sender cells	0.41 nM s^{-1}	N/A
α_{12}	Production of 3OC12-HSL in sender cells	0.0067 nM s^{-1}	N/A
D_6	Diffusion of 3OC6-HSL	9.91×10^{-10} m^2 s^{-1}	3.79×10^{-10} m^2 s^{-1}
D_{12}	Diffusion of 3OC12-HSL	1.56×10^{-10} m^2 s^{-1}	1.99×10^{-10} m^2 s^{-1}

F.2 Re-characterizing the pLas81 response

Due to the inaccuracy of the simplified model with respect to the YFP measurements in pR33S175 (Fig. S8B, bottom panel), we decided to use an alternative model of the pLas81 response to 3OC12-HSL. We used a Hill function defined and parameterized as

$$h_{81}([C_{12}]) = \frac{a_1[C_{12}]^n + a_0 K^n}{[C_{12}]^n + K^n}, \tag{35}$$

which enabled a close fit to the 3OC12-HSL response (Figure S11A). The optimized parameter values were $a_1 = 9.0701$ RPU, $a_0 = 0.1945$ RPU, $K = 111$ nM, $n = 0.96$.

We then used the Hill function model of the pLas81 response, in combination with the original simplified model of the pLux76 response, to characterize the diffusion and degradation of HSL molecules. The characterization used experimental data from an assay that was similar to the setup in Figure 3D of the main text. However, here the HSL was pipetted into the left and rightmost grid cells, instead of using HSL sender cells. In this way, we were able to parameterize system (29) without needing to also fit parameters for the rate of HSL synthesis. The model was able to fit well to the early increase in CFP and YFP expression, though poorly reproduced the levelling off and decline in CFP signal after approximately 15 hours (Figure S11B,C). This indicated that the simplistic approach of modelling HSL degradation proportional to its local concentration was possibly insufficient. Further refinements could include using HSL-specific degradation rates, and/or making HSL degradation proportional to cell density.

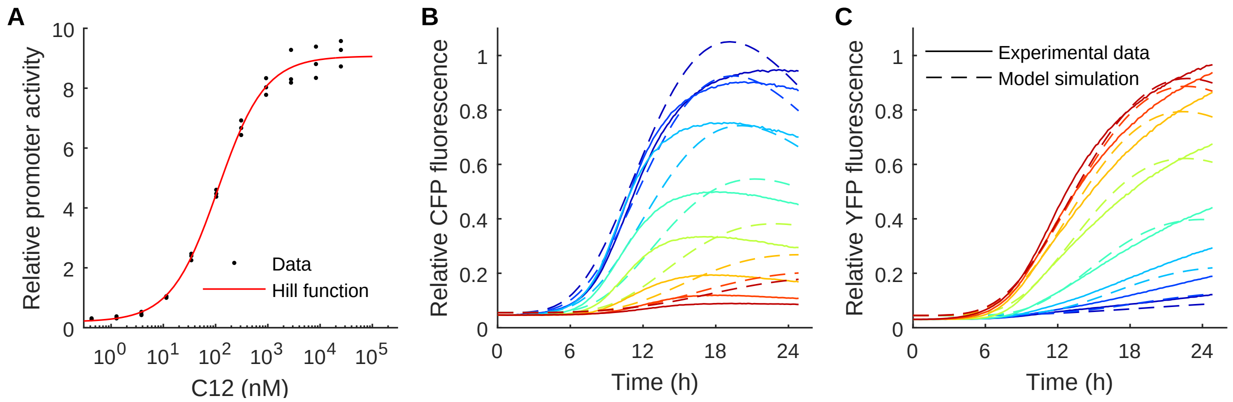

Figure S11: Using a Hill function to model the pLas81 response of pR33S175. (**A**) The parameters of the Hill function were optimized using `fminsearch` to solve a least-squares cost function in Matlab. The optimal values were $a_1 = 9.07$, $a_0 = 0.19$, $K = 111$ and $n = 0.96$. (**B,C**) The HSL diffusion rates and an HSL degradation rate were fit to experimental measurements of a spatial receiver assay. The assay was similar to the setup in Figure 3D, except that HSL was pipetted at a concentration of 50 μM into the left- and rightmost grid cells, instead of using HSL senders. The optimal parameters were determined to be $D_6 = 3.79 \times 10^{-10}$ m^2 s^{-1}, $D_{12} = 1.99 \times 10^{-10}$ m^2 s^{-1}, and $\gamma_{HSL} = 3.32 \times 10^{-5}$ s^{-1}.

F.3 Characterization of relay sender devices

To characterize the LuxI and LasI enzymes in the spatial assay system, we measured double receiver cells responding to diffusion of LuxI-synthesized 3OC6-HSL emanating from 3OC12-HSL-inducible cells (pLas81-LuxI; Figure 4A) and diffusion of LasI-synthesized 3OC12-HSL emanating from 3OC6-HSL-inducible cells (pLux76-LasI; Figure 4B). When using the simplified model for the pLux76 and pLas81 responses, we found that the YFP channel data could not be accurately modelled (not shown). To prevent these inaccuracies from biasing the characterization of LuxI and LasI, we used the Hill function model for pLas81 and the corresponding HSL diffusion and degradation rates characterized in Figure S11. Comparison of this model setup with the experimental data is shown in Figure 4C,D of the main text.

The model for the relay sender cells was similar to the model of the double receiver cells (Eqn. 29). The crucial difference here is that LuxI/LasI are explicit dynamic quantities. By denoting I_6 as the non-dimensionalized concentration of LuxI and I_{12} as the non-dimensionalized concentration of LasI, we can write down a model for the relay sender cells as:

$$[N_S](t) = K_S e^{\log\left(\frac{c_0}{K_S}\right) e^{-r_S . t}} \quad (36a)$$

$$[C_i] = C_0 \quad (36b)$$

$$I_j = \alpha_{Ij} f - d_{Ij} I_j \quad (36c)$$

$$\frac{\partial [C_j]}{\partial t} = b_j \frac{I_j^{n_{Ij}}}{1 + I_j^{n_{Ij}}} - \gamma_{\text{HSL}}[C_j] + D_j \frac{\partial^2 [C_j]}{\partial x^2} \quad (36d)$$

$$\frac{\partial [\text{iCFP}]}{\partial t} = \alpha_C [N].f_{76}(R, S, [C_6], [C_{12}]) - (\gamma_{\text{FP}} + \mu)[\text{iCFP}] \quad (36e)$$

$$\frac{\partial [\text{iYFP}]}{\partial t} = \alpha_Y [N].h_{81}([C_{12}]) - (\gamma_{\text{FP}} + \mu)[\text{iYFP}] \quad (36f)$$

$$\frac{\partial [\text{iCFP}]}{\partial t} = \mu[\text{iCFP}] - \gamma_{\text{FP}}[\text{CFP}] \quad (36g)$$

$$\frac{\partial [\text{iYFP}]}{\partial t} = \mu[\text{iYFP}] - \gamma_{\text{FP}}[\text{YFP}] \quad (36h)$$

where $(i, j, f) = (6, 12, f_{76})$ for pLux76-LasI and $(i, j, f) = (12, 6, h_{81})$ for pLas81-LuxI. According, C_i denotes the concentration of the input HSL and C_j denotes the concentration of the induced HSL.

Inferring cell growth parameters

As before, the cell growth parameters were inferred separately, since these could be extracted from the RFP data. The method of Appendix F.1 was used, but with separate growth curves for the relay senders cells and double receiver cells, and also for each concentration of HSL input. For each input concentration, the relay sender and double receiver models were fit to the data simultaneously. The cell densities were scaled relative to the double receiver cells, with a relative carrying capacity inferred for the relay senders. By fitting simultaneously, we could use the same RFP degradation rate and the same model-data scaling (c.f. Eqn. 32).

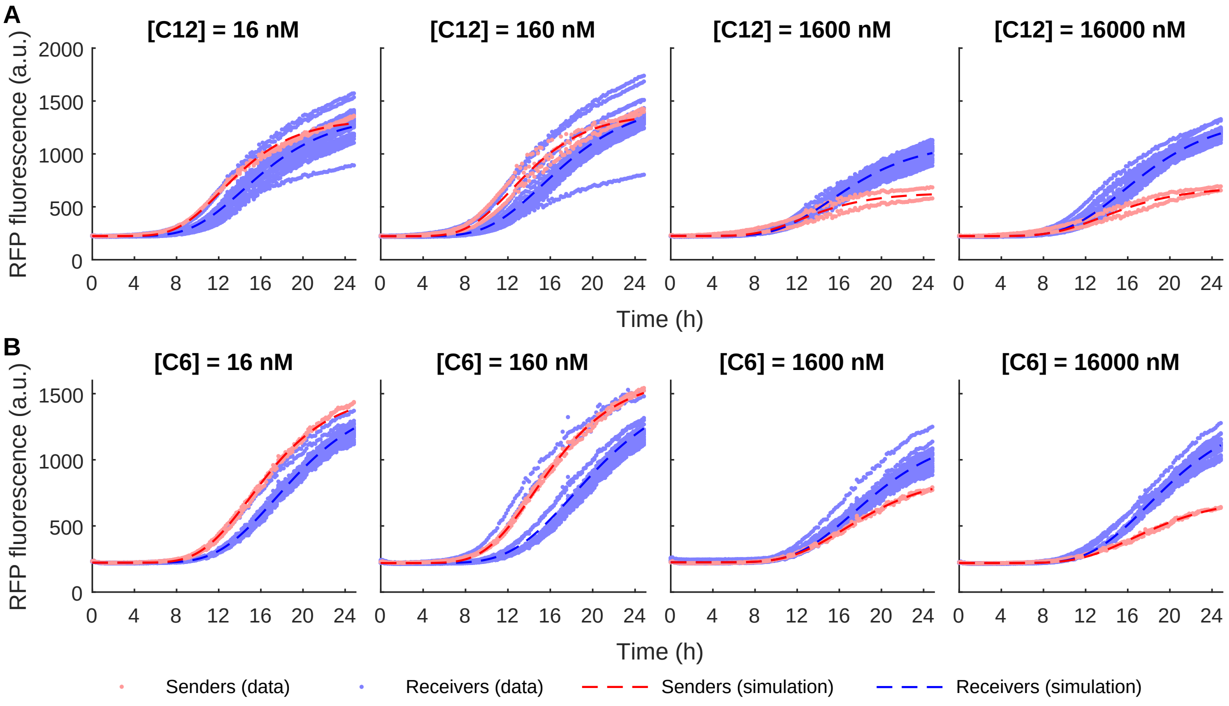

Figure S12: Characterization of cell density using RFP fluorescence data. Characterization of cell growth in experiments measuring double receiver cells and (**A**) pLas81-LuxI relay sender cells or (**B**) pLux76-LasI sender cells, in response to 3OC6-HSL or 3OC12-HSL as indicated above each panel. RFP measurements are indicated by the dots, with the model indicated by dashed lines. Red lines/symbols indicate relay sender cells, while blue lines/symbols indicate double receiver cells.

Inferring relay enzyme parameters

The remaining parameters were inferred from the data in Fig. 4A and 4B of the main text. We used the same approach to that taken above, using `fminsearch` to minimize a cost function with a scale parameter λ that is calculated using linear regression. The LuxI and LasI components were characterized separately however, fitting a_{LuxI}, b_6 and n_{LuxI} to the data in Fig. 4A, and then a_{LasI}, b_{12} and n_{LasI} to the data in Fig. 4B (see Table S9 the optimized values). The cost function combined data for both the CFP and YFP measurements, and had an equivalent form to Eqn. 34. The primary responses of each relay circuit are compared in Fig. S13, while the secondary responses are compared in Fig. 4C,D in the main text.

Table S9: Summary of optimized parameters for the spatial model of relay senders.

Parameter	Description	Value
a_{LuxI}	Rate of luxI synthesis	7.63×10^{-5} nM s^{-1}
a_{LasI}	Rate of lasI synthesis	8.00×10^{-5} nM s^{-1}
d_I	Rate of LuxI/LasI degradation	10^{-3} s^{-1}
b_6	Rate of 3OC6-HSL synthesis	0.47
b_{12}	Rate of 3OC12-HSL synthesis	0.096
n_{LuxI}	Hill coefficient for LuxI-mediated 3OC6-HSL synthesis	3.21
n_{LasI}	Hill coefficient for LasI-mediated 3OC12-HSL synthesis	1.84

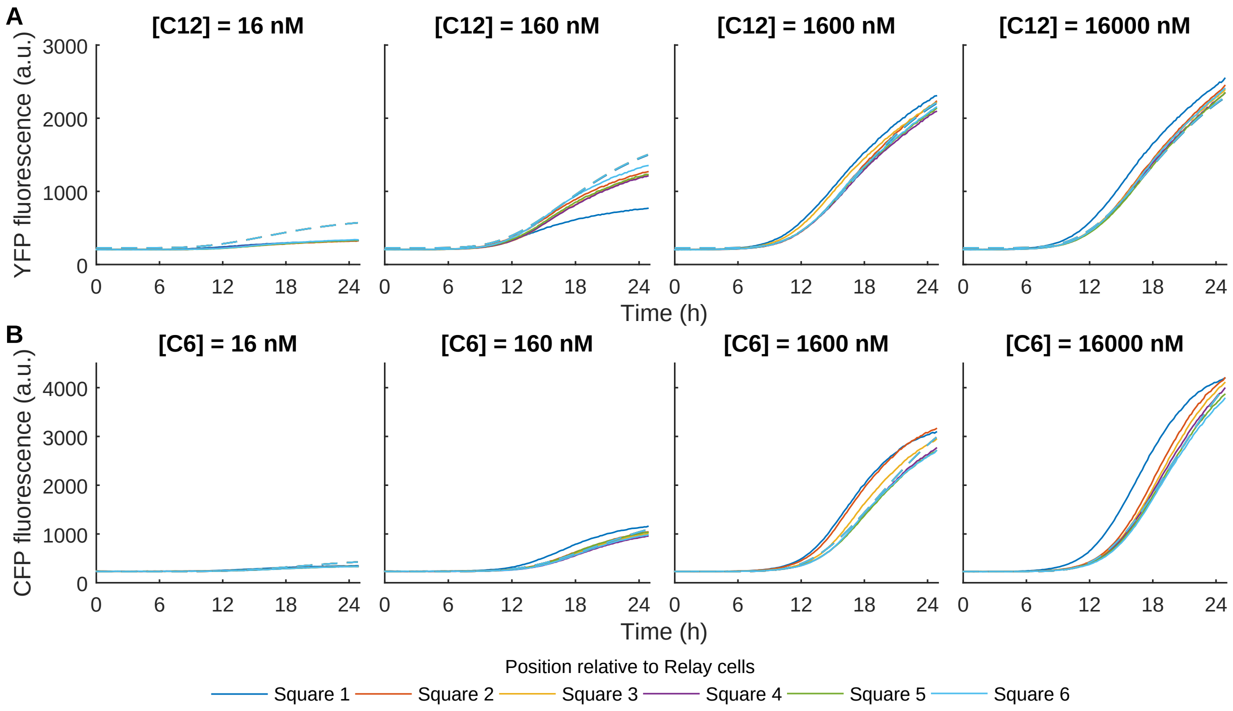

Figure S13: Double receiver cells respond to relay cells in a concentration-dependent manner. Comparison of the model against experimental measurements for double receiver cells responding to (**A**) 3OC12-HSL or (**B**) 3OC6-HSL. The data shown are from the same experiment as shown in Figure 4 of the main text, here reporting the alternative fluorescence channel. RFP measurements are indicated by the solid lines, with the model indicated by dashed lines. Each color indicates a different grid cell, numbered by their distance from the relay sender cells.

F.4 Simulation of relay cells in a checkerboard arrangement

Simulations were carried to determine whether it was possible to predict complex 2-dimensional spatiotemporal behaviors, using parameter values calibrated as described above. By placing the two relay cell types in a checkerboard arrangement, as reported in Fig. 5D, a positive feedback is created, though diffusion is required to transduce the feedback signals. However, in the *induced* variant, both cell types are able to communicate with one another with short range cell-cell communication.

The model was initialized in a checkerboard pattern, with both cell types in the central 8x8 grid cells, then simulated as a 2-dimensional PDE with zero-flux boundary conditions. With the parameters quantified in Tables S8 and S9, we found that the positive feedback was not sufficiently strong to exhibit *inducible* behavior, and stabilized at a spatially uniform equilibrium with low levels of HSL and CFP/YFP. We found that by increasing the rate of LuxI/LasI transcription by a factor 5 or greater, that the simulations did exhibit inducible behavior (Fig. EV3A, Movie S2). Therefore, the first observation is that the model predictions are qualitatively incorrect.

Our interpretation is that diffusion stabilizes the system at low levels unless LuxI/LasI is strong enough to force the system to the higher equilibrium. To illustrate this bifurcation, we simulated the positive feedback loop in LuxI/LasI/3OC6-HSL/3OC12-HSL in spatially homogeneous conditions (i.e. without diffusion). We found that a bifurcation exists when increasing LuxI/LasI transcription rates past a scale factor 1.7, switching from a low to a high equilibrium in all components (Fig. EV3B). We suggest that when there is diffusion, HSL dissipates from the central region, which contributes an opposing force against the positive feedback, thus moving the position of the bifurcation. This helps to explain why a larger increase in LuxI/LasI transcription would be needed to reproduce the inducible behaviors.

References

[1] T. J. Rudge, J. R. Brown, F. Federici, N. Dalchau, A. Phillips, J. W. Ajioka, and J. Haseloff, “Characterization of intrinsic properties of promoters.,” *ACS Synth Biol*, Oct 2015.

[2] B. Yordanov, N. Dalchau, P. K. Grant, M. Pedersen, S. Emmott, J. Haseloff, and A. Phillips, “A computational method for automated characterization of genetic components.,” *ACS Synth Biol*, vol. 3, pp. 578–588, Aug 2014.

[3] H. M. Salis, E. A. Mirsky, and C. A. Voigt, “Automated design of synthetic ribosome binding sites to control protein expression.,” *Nat Biotechnol*, vol. 27, pp. 946–950, Oct 2009.

[4] B. Wang, R. I. Kitney, N. Joly, and M. Buck, “Engineering modular and orthogonal genetic logic gates for robust digital-like synthetic biology.,” *Nat Commun*, vol. 2, p. 508, 2011.

[5] A. Tamsir, J. J. Tabor, and C. A. Voigt, “Robust multicellular computing using genetically encoded nor gates and chemical 'wires'.,” *Nature*, vol. 469, pp. 212–215, Jan 2011.

[6] R. Daniel, J. R. Rubens, R. Sarpeshkar, and T. K. Lu, “Synthetic analog computation in living cells.,” *Nature*, vol. 497, pp. 619–623, May 2013.

[7] M. Pedersen and A. Phillips, “Towards programming languages for genetic engineering of living cells,” *Journal of the Royal Society Interface*, April 2009.

[8] M. H. Zwietering, I. Jongenburger, F. M. Rombouts, and K. van 't Riet, “Modeling of the bacterial growth curve.,” *Appl Environ Microbiol*, vol. 56, pp. 1875–1881, Jun 1990.
