# Supplementary material for: Orthogonal intercellular signaling for programmed spatial behavior
Source: Mol Syst Biol. 2016 Jan 26;12(1):849. doi: 10.15252/msb.20156590 (PMC4731010; doi:10.15252/msb.20156590)
Supplement: Supplementary file 2 — Expanded View Figures PDF [file MSB-12-849-s002.pdf]

Expanded View Figures

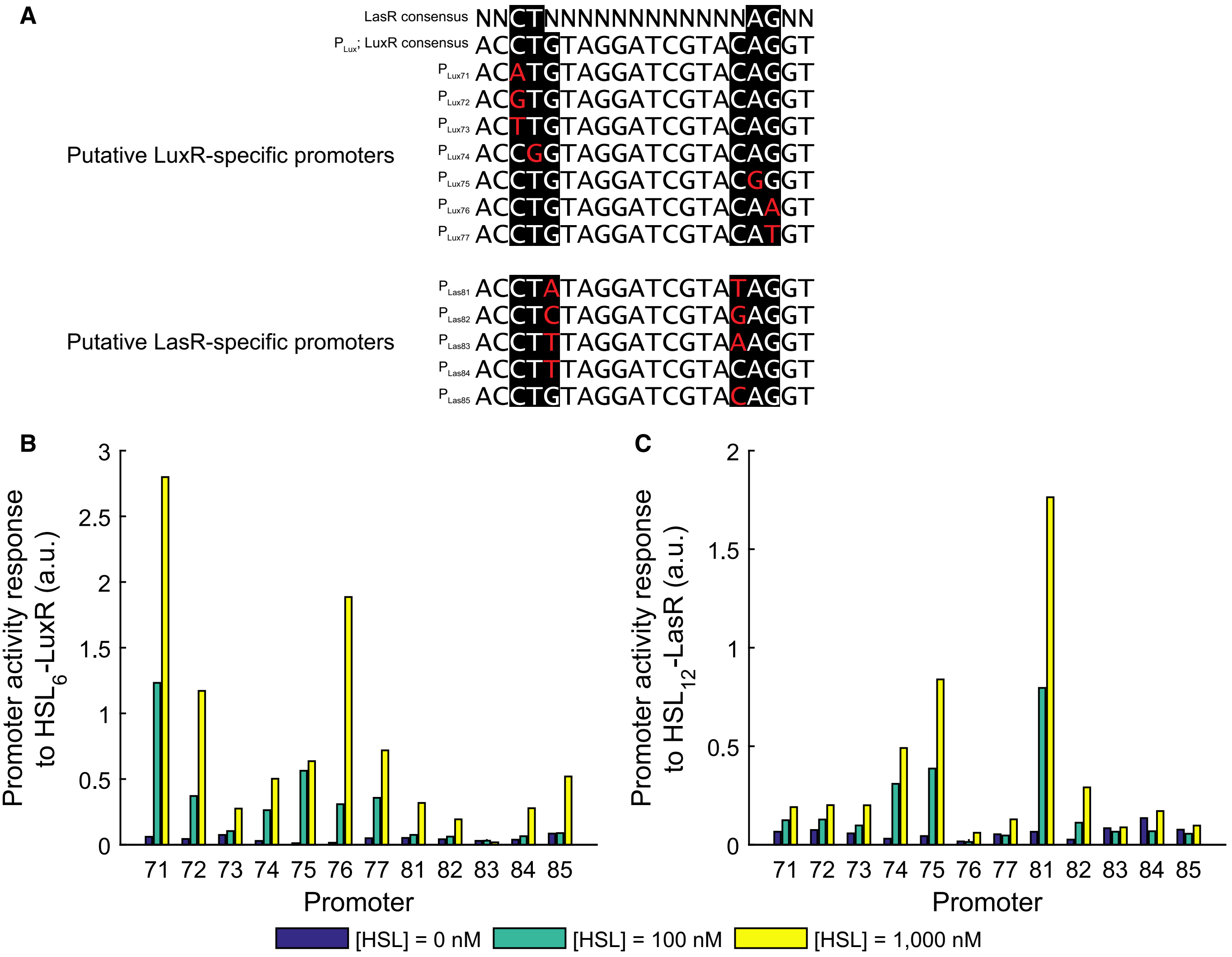

Figure EV1. Evaluation of rationally designed putative LuxR- or LasR-specific mutations in pLux.

A Promoter sequences. Consensus-binding sequences are highlighted in black while specific mutations are in red.

B Activity of mutant promoters (deYFP/dOD) in the presence of strong expression of receiver proteins with reference promoter Bba J23101 (identical to pR0011LL123* except for the promoter driving eYFP) measured in 3OC6-HSL concentrations as shown in color code.

C Activity as in (B) measured in 3OC12-HSL concentrations as shown in color code.

 © 2016 The Authors

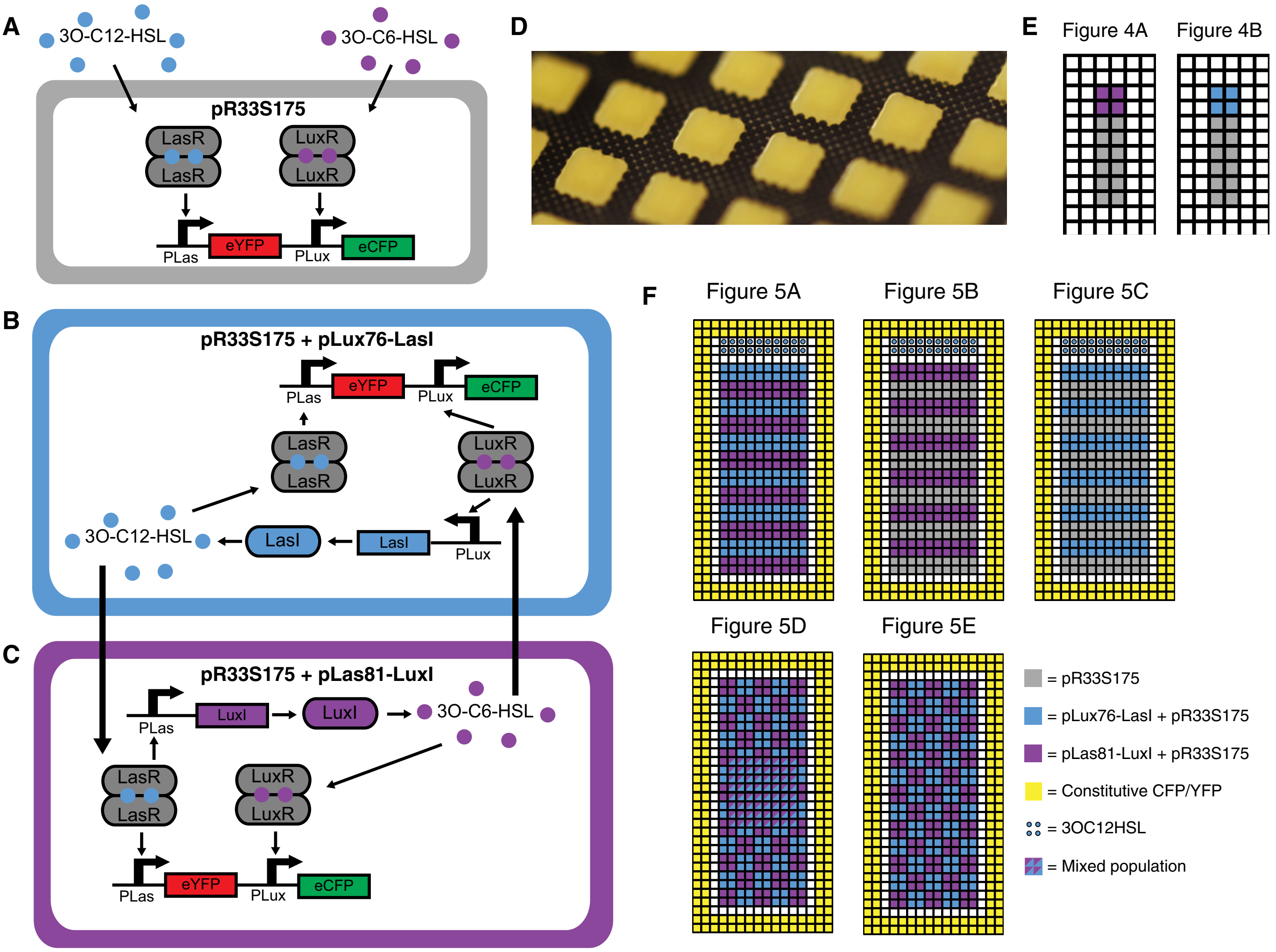

Figure EV2. Cell types and layout of populations used in solid culture assays.

A–C Genotypes and regulatory interactions for each of the cell types used in solid culture assays. (A) pR33S175 responds to exogenous 3OC6HSL by producing CFP and to 3OC12HSL by producing YFP. (B) Addition of pLux76-LasI causes the production of 3OC12HSL in response to 3O-C6-HSL. (C) Addition of pLas81-LuxI causes the production of 3OC6HSL in response to 3OC12HSL

D Photograph of populations grown on membranes printed with hydrophobic grids.

E Initial layout of populations for experiments presented in Fig 4.

F Initial layout of populations for experiments presented in Fig 5.

© 2016 The Authors

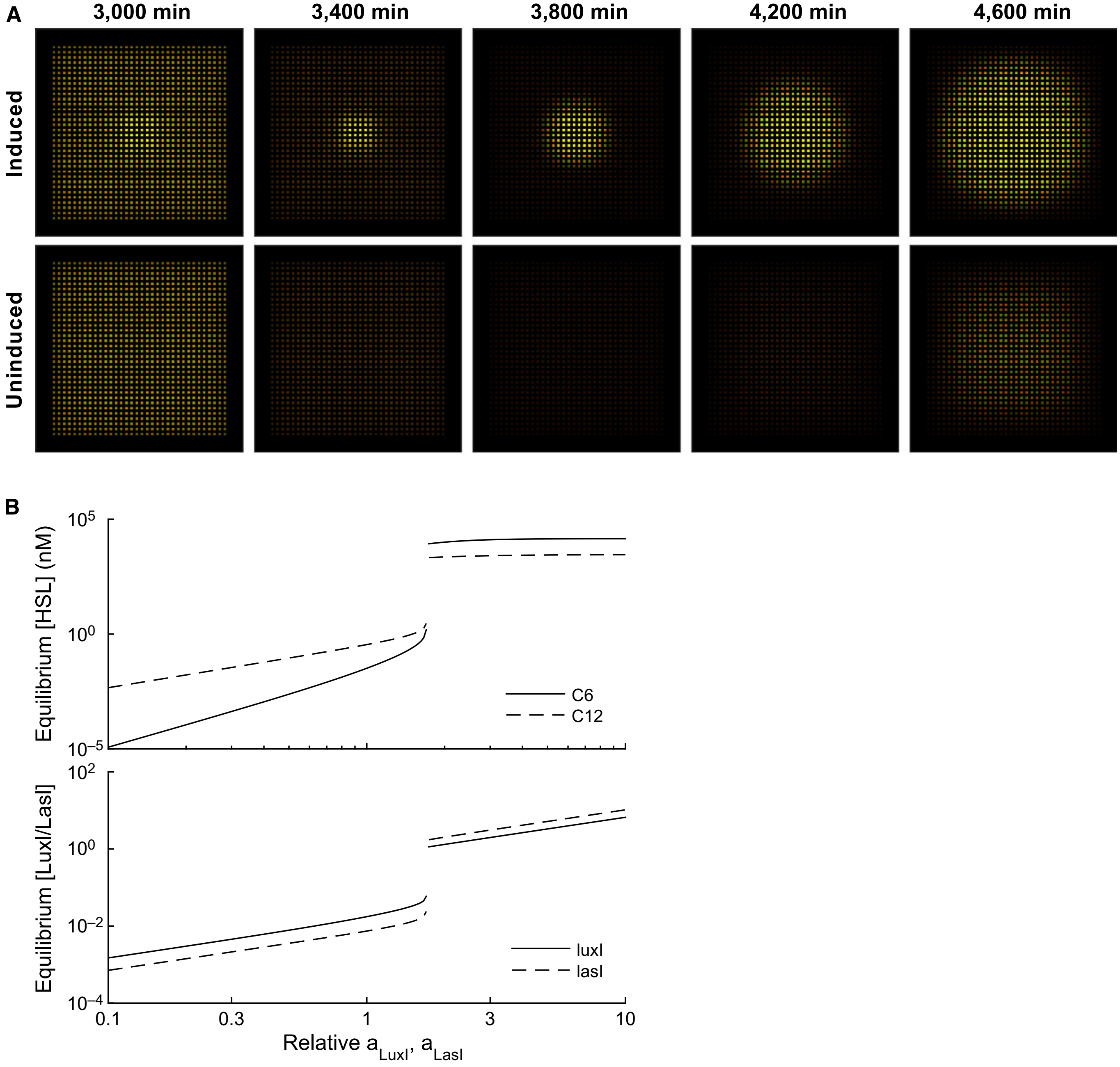

Figure EV3. Model analysis of relay cells in a checkerboard arrangement.

A Simulation of relay cells in a checkerboard arrangement. Simulations were carried out as described in Appendix F.4. Shown are simulations where either both cell types are initialized in the central 8 × 8 grid cells (induced), or not (uninduced; see Fig EV2F).

B Increasing LuxI/LasI transcription induces a bifurcation from low to high levels. A spatially homogeneous model was simulated assuming a direct feedback between the pLux76-LasI and pLas81-LuxI modules. The model parameters were initialized at inferred values; then, the production rates of both LuxI and LasI were multiplied by the scale factor indicated on the horizontal axis.

© 2016 The Authors
