# Supplementary material for: Orthogonal intercellular signaling for programmed spatial behavior
Source: Mol Syst Biol. 2016 Jan 26;12(1):849. doi: 10.15252/msb.20156590 (PMC4731010; doi:10.15252/msb.20156590)
Supplement: Supplementary file 3 — Movie EV1 [file MSB-12-849-s008.zip › Movie_EV1_legend.rtf]

Movie EV 1Images taken every 10 minutes for 4000 minutes.  The eCFP channel is green; the eYFP channel is red, and the mRFP1 channel is blue.  Top left corresponds with figure 5C.  Top right corresponds with figure 5B.  Bottom right corresponds with figure 5A.  Bottom left is double receiver with empty vector induced with C12.
