# Supplementary material for: Orthogonal intercellular signaling for programmed spatial behavior
Source: Mol Syst Biol. 2016 Jan 26;12(1):849. doi: 10.15252/msb.20156590 (PMC4731010; doi:10.15252/msb.20156590)
Supplement: Supplementary file 4 — Movie EV2 [file MSB-12-849-s009.zip › Movie_EV2_legend.rtf]

Movie EV2 Images taken every 10 minutes for 5750 minutes.  The eCFP channel is green and the eYFP channel is red.  Left corresponds with figure 5E. Right corresponds with figure 5D.
